# Assessing the Heterogeneous Treatment Effects of Glucocorticoids in Infants and Toddlers with Severe Pneumonia

**DOI:** 10.3390/biomedicines13102333

**Published:** 2025-09-24

**Authors:** Zhoumeng Ying, Haiyan Ge, Wei Han, Ge Hu, Zhenchen Zhu, Jinhua Wang, Lan Song, Dong Qu, Zhengyu Jin

**Affiliations:** 1 Department of Radiology, State Key Laboratory of Complex Severe and Rare Diseases, Peking Union Medical College Hospital, Chinese Academy of Medical Sciences and Peking Union Medical College, Beijing 100005, China; 2 Department of Critical Care Medicine, Capital Center For Children’s Health, Capital Medical University, Beijing 100020, China; 3 Department of Epidemiology and Health Statistics, Institute of Basic Medicine Sciences, School of Basic Medicine, Chinese Academy of Medical Sciences and Peking Union Medical College, Beijing 100005, China; 4 Theranostics and Translational Research Center, National Infrastructures for Translational Medicine, Institute of Clinical Medicine, Peking Union Medical College Hospital, Chinese Academy of Medical Sciences and Peking Union Medical College, Beijing 100005, China

**Keywords:** pneumonia, pediatrics, glucocorticoids, treatment effect heterogeneity, supervised machine learning

## Abstract

**Background**: Community-acquired pneumonia (CAP) is a leading cause of pediatric hospitalizations and a risk factor for chronic respiratory conditions. Glucocorticoids (GCs) are used as adjunctive therapy to reduce inflammation, but their efficacy in infants and toddlers remains unclear. **Method**: A retrospective study of 1116 infants and toddlers with severe CAP was conducted, using causal forest to estimate individual treatment effects (ITEs), with the duration of intensive care unit (ICU) stay as the outcome. Patients were stratified based on ITEs to investigate the heterogeneous treatment effect (HTE) and identify responders. Generalized linear models validated the HTE across subclasses, followed by comparative analyses to characterize responders. Variable importance was assessed using the causal model, and Shapley additive explanations (SHAP) quantified each variable’s contribution to the ITE. Analysis was also performed in mechanically ventilated patients (MV group). **Results**: GCs demonstrated significant HTE. Older patients and those with elevated inflammation markers showed better responses, whereas no such benefit was observed in respiratory syncytial virus-infected patients. These subgroups experienced shorter ICU stays both in the whole cohort (β = −0.16, *p* < 0.001) and MV group (β = −0.34, *p* < 0.001), and shorter ventilation duration was observed in the MV group (β = −0.35, *p* < 0.001). Age and the anion gap were key predictors of ITEs. SHAP analysis revealed a positive correlation between age and GC effectiveness. **Conclusions**: Significant heterogeneity in GC treatment effects exists among infants and toddlers with severe CAP, highlighting the need for optimization of GC use in this population.

## 1. Introduction

Pneumonia is among the leading causes of hospitalization in children, with an incidence ranging from 34 to 40 cases per 1000 children in Europe and North America [1]. Long-term studies have shown that pneumonia in early childhood is linked to a higher risk of chronic respiratory conditions later in life, such as bronchitis, and recurrent pneumonia [2]. Since early childhood is a crucial period for lung growth and development [3], providing effective and precise treatments for this vulnerable group is essential. Glucocorticoids (GCs) are used as adjunctive therapy to modulate inflammation in CAP [4]. However, their effectiveness among this vulnerable group remains unclear.

In adults, seven randomized controlled trials (RCTs) have suggested that GC treatment could benefit patients with CAP [5,6,7,8,9,10,11], but two additional RCTs and a meta-analysis of 16 trials found no significant impact on mortality [12,13,14]. Additionally, stratified subgroup analyses have shown that GCs are effective only in severe cases, with no significant benefit in non-severe cases, suggesting a marked heterogeneity in the treatment effect (HTE) of GCs among CAP patients [15,16]. Research on systemic GCs for CAP in the pediatric population is limited [17,18,19,20,21,22,23,24,25,26]. Only two retrospective studies [24,25] and two small-scale RCTs [20,26] focused on pediatric CAP patients, indicating that the efficacy of GCs in this population remains largely unexplored, particularly in infants and toddlers. Furthermore, no research has addressed the HTE of GCs in pediatric CAP patients. In addition to unclear efficacy, GC treatment is associated with a 1.4- to 2.2-fold increased risk of gastrointestinal bleeding and sepsis following therapy, indicating the importance of precision in GC use to minimize unnecessary steroid-related adverse effects [27].

HTE is characterized as nonrandom, explicable variability in the direction and magnitude of treatment effects [28]. Various machine learning (ML) algorithms have recently been employed for HTE analysis [29]. Among these, the causal forest model is a supervised method that uses an ensemble of decision trees specifically optimized to detect HTEs and estimate the individual treatment effect (ITE) [30,31]. This approach has been demonstrated effective in identifying ITE in diverse clinical data [32,33,34,35,36,37].

Given the significant research gap concerning the effects of GCs in infants and toddlers with severe CAP, we conducted a retrospective cohort study in the pediatric intensive care unit (PICU) and employed the causal forest model to explore the efficacy and HTE of GCs. The study objectives were to explore the therapeutic efficacy and HTE of GCs, identify GC responders, and characterize their features. These findings will increase the precision of GC therapy, thereby optimizing treatment strategies for these vulnerable population.

## 2. Materials and Methods

This study and all its protocols were approved by a tertiary children’s specialized hospital (approval number: SHERLLM2023022, approval date: 3 March 2023), written informed consent was not required for this study due to the retrospective nature.

### 2.1. Patient Population and Study Design

This retrospective study analyzed data from all consecutive infants and toddlers admitted to the PICU of a tertiary children’s specialized hospital for severe CAP from January 2016 to December 2023 (Supplementary Figure S1). The dataset was locked on 20 March 2024 prior to analysis. The inclusion criteria were: (1) patients with age greater than 28 days and up to 3 years, (2) and those diagnosed with severe pediatric CAP requiring ICU care. The diagnosis and severity of pneumonia were determined based on the 2019 pediatric CAP guideline [38] and independently verified by a senior physician specialized in pediatric critical care. The diagnostic details and classification criteria are detailed in the Supplementary Methods S1 and S2.

The exclusion criteria included: (1) those with a history of aspiration or diagnosed with aspiration pneumonia, (2) those with immunocompromising or chronic medical conditions predisposing to severe or recurrent pneumonia, including immunodeficiency, malignancy, chronic lung disease, cystic fibrosis, congenital anomalies of the airways or lung, severe malnutrition, and congenital heart disease, and (3) those who had received systemic steroids within the last 30 days. (4) those who experienced mortality during hospitalization.

### 2.2. Covariates, Exposure, and Outcomes

The covariates for this study were selected to adjust for confounding, based on their role as common causes of both the treatment and outcome, as depicted in our causal framework (Supplementary Figure S2) and detailed in Supplementary Methods S3. The final set of covariates included demographic characteristics, complete blood count, liver and renal function tests, C-reactive protein (CRP), blood gas analyses, and etiological tests at ICU admission. Data collection was facilitated through a digital hospital information system (HIS). The normal reference ranges for pediatric laboratory values were defined according to two industry standards [39,40]. And the classification of etiological infection types, laboratory tests, and preexisting comorbidities was detailed in Supplementary Methods S4–S6.

The exposure was defined as the administration of at least three-days of adjunctive systemic GCs, either orally or intravenously, including dexamethasone, hydrocortisone, methylprednisolone, prednisolone, or prednisone. To standardize the dosage, all GC doses were converted to methylprednisolone-equivalent doses, with the first-day dose serving as an indicator of treatment intensity. The specific GC types administered were summarized in Supplementary Table S1. The interval between ICU admission and the initiation of GC therapy was also recorded. A history of GC use was also obtained from the HIS.

The primary outcome of this study was the duration of ICU stay. For the subgroup analysis, we focused on patients who required mechanical ventilation (the mechanical ventilation [MV] group). In this subgroup, alongside the primary outcome, the duration of MV was evaluated as a secondary outcome.

### 2.3. Model Construction and HTE Analysis

**Figure 1:**
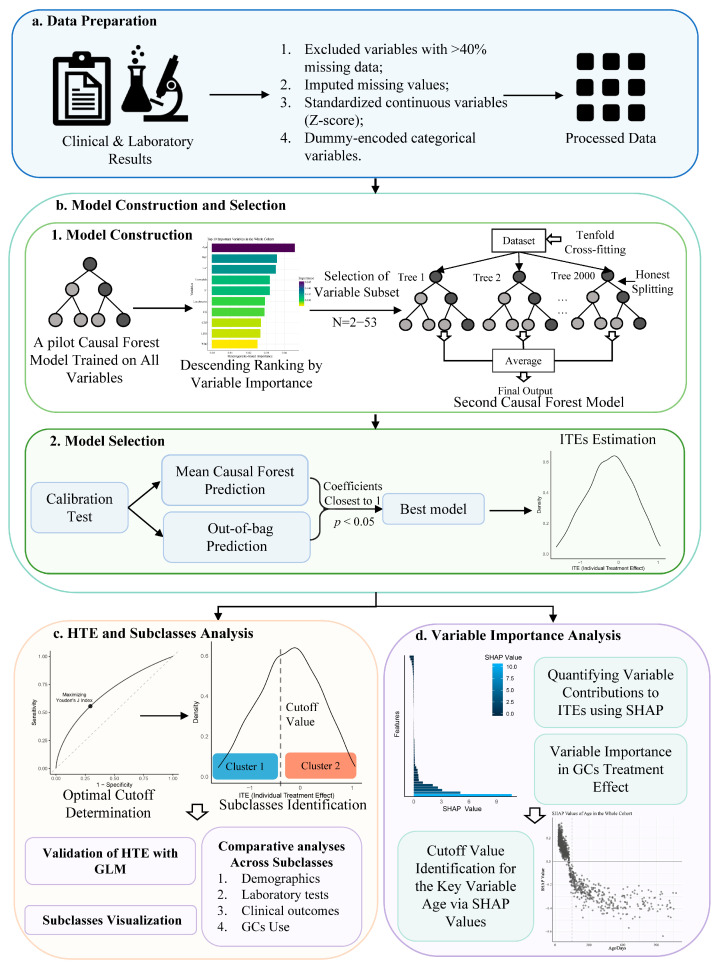
Overview of the Model Construction and Analysis Workflow. (**a**) Data Preparation: Clinical and laboratory results were collected for all patients. Variables with over 40% missing data were excluded, and missing laboratory data were imputed. Continuous variables were standardized using z-scores, while categorical variables were dummy coded. (**b**) Model Construction and Selection: (1) Model construction: A causal forest model trained on the all variables was built. Variable importance was assessed with the initial model, followed by ranking and selecting variables for a second causal forest model. The second model was constructed with 2000 trees, optimized through tenfold cross-validation. And the final model was selected by calibration tests. (2) Model selection: Calibration was evaluated using the best linear predictor for the true conditional average treatment effects. A well-calibrated model was indicated by a statistically significant coefficient near one for both the mean and differential prediction. The model with the best performance was selected as the final causal forest model. And the Individual treatment effects (ITEs) for GCs were calculated. (**c**) HTE and Subclass Analysis: Receiver operating characteristic curves were plotted, and optimal ITE cutoff values were identified using Youden’s J Index. Patients were grouped into two clusters based on ITEs, and multivariable generalized linear models were used to evaluate GC effects within each subclass, validating the heterogeneity of treatment effect among the patients. Additionally, comparative analyses across the subclasses were conducted. (**d**) Variable Importance Analysis: Shapley additive explanation values were computed to quantify the contribution of each variable to ITEs at the individual level. And the variable importance was calculated based on split occurrences in the causal forest model. Then the influence of age on the ITEs was analyzed separately to identify the cutoff value. Abbreviations: ITEs: individual treatment effects, HTE: the heterogeneous treatment effect, GLM: generalized linear model, GCs: glucocorticoids, SHAP: Shapley additive explanations.

The overall workflow chart is illustrated in Figure 1.

#### 2.3.1. Data Preparation

Variables with more than 40% missing data were excluded to avoid unreliable imputations [41]. Supplementary Table S2 lists the excluded variables and their missingness. Then missing laboratory data were addressed through multiple imputation with classification and regression trees via the ‘mice’ package. Continuous variables were standardized via z-scores, whereas categorical variables were encoded with dummy variables.

#### 2.3.2. Model Construction and Selection

We estimated ITEs using an augmented inverse probability weighting (AIPW) causal forest with cross-fitting. The model was constructed from 2000 trees via an honest splitting approach along with tenfold cross-fitting, while hyperparameters were optimized through tenfold cross-validation based on minimizing out-of-bag (OOB) prediction error [30,42]. Initially, a pilot causal forest model was used to estimate variable importance and rank variables in order of importance, guiding the selection of variables for the second model. Next, calibration tests, using the best linear predictor of conditional average treatment effects (CATE) from OOB predictions, determined the number of variables for the second model. A coefficient close to one for both the mean prediction and the differential prediction demonstrated strong calibration and effective capture of HTE [30,31,43]. The model with best performance was selected as the final model. More details about the model calibration can be found in Supplementary Methods S7.

#### 2.3.3. HTE and Subclasses Analysis

The causal forest model’s performance was further validated using the calibration plot. Following this validation, and for the exploratory purpose of characterizing responders’ profiles, we then divided the continuous ITEs into two subclasses. An optimal ITE cutoff was identified using Receiver operating characteristic (ROC) curves comparing GC usage with ITEs via the Youden’s J Index. Based on these cutoffs, patients were grouped into Cluster 1 (lower ITEs, shorter ICU stay, better response) and Cluster 2 (higher ITEs, longer ICU stay, poorer response). Multivariable generalized linear models (GLMs) with a Gamma distribution and log link were used to assess the impact of GCs in each cluster. Confounders were selected based on the Akaike information criterion [44].

#### 2.3.4. Variable Importance Analysis

Variable importance was quantified by aggregating the weighted occurrences of each variable’s splits across depths in the causal forest model [43]. Shapley additive explanations (SHAP) were also calculated based on ITEs, quantifying each variable’s contribution to ITEs at the individual patient level [45,46].

### 2.4. Sensitivity Analysis

To assess the robustness of our missing-data strategy, we conducted two sensitivity analyses in addition to the primary multiple imputation approach. First, a complete-case analysis restricted to patients with no missing covariates was performed. Second, a missing-indicator method was applied, in which binary indicators were added for missing values and continuous variables were set to cohort medians.

To further assess the robustness of the observed HTEs, we performed an additional sensitivity analysis using an alternative stratification approach based on ITE quartiles (K = 4), in contrast to the primary binary classification derived from Youden’s J index. Patients were grouped into four quartiles according to their estimated ITEs, and multivariable GLMs were applied within each quartile to evaluate the effect of GC therapy on ICU and MV durations. To further support the validity of the responder profiles identified in the primary two-group stratification, we further compared key clinical and laboratory features between Quartiles 1 and 4, representing the most divergent treatment response groups. To assess the robustness of the ATE derived from our primary causal forest model, we conducted a sensitivity analysis using overlap weighting with inverse probability weights derived from a logistic regression including all baseline covariates. Covariate balance before and after weighting was assessed using standardized mean differences (SMDs). The overlap-weighted ATE was then estimated using weighted linear regression.

### 2.5. Statistical Analysis

For categorical variables, group comparisons were performed via the chi-square test, whereas normally distributed continuous data were analyzed via the t test, and nonnormally distributed data were assessed via the Wilcoxon rank-sum test. To account for multiple comparisons, the Benjamini–Hochberg procedure was employed to adjust *p* values for multiple comparisons, controlling the false discovery rate.

All the statistical analyses were conducted via R (version 4.3.1; R Foundation for Statistical Computing, Vienna, Austria). The ‘mice’ package (version 3.14.0) was used for multiple imputation. The ‘grf’ (version 2.3.1) and ‘iml’ (version 0.11.4) packages in R were employed to construct the causal forest model and compute SHAP values [42,47]. Calibration tests and plots were performed to evaluate the performance of the causal forest model [30,31,43,48]. More details about the model calibration can be found in Supplementary Methods S7. For transparency, we additionally report overlap diagnostics in the Supplementary Figure S3, including OOB C-statistic of the cross-fitted treatment model, SMDs before and after forest-based weighting, and propensity-score overlap. A two-sided *p* < 0.05 was considered statistically significant throughout the study. The R scripts for the causal forest analysis were made available at https://github.com/zhoumeng1022/HTE_Severe_Pneumonia (commit a1b2c3d, accessed on 27 August 2025).

## 3. Results

### 3.1. Participant Characteristics

A total of 1116 patients (median age: 82.50 days [IQR: 51.00,200.00]) were included in this study (Supplementary Table S3), with 273 receiving GC therapy. Patients treated with GCs were older (median age [IQR]: 247.00 days [92.00,511.00] vs. 69.00 days [48.00,122.00], *p* < 0.001) and exhibited a greater likelihood of requiring MV (56.41% vs. 25.39%, *p* < 0.001), and longer duration of ICU stay (median [IQR]: 6.96 days [5.71,9.75] vs. 5.83 days [4.50,7.92], *p* < 0.001) than non-GC users.

### 3.2. Subclass Identification and Intergroup Analysis in the Whole Cohort

The final optimal model of the whole cohort demonstrated good calibration, with a coefficient of 1.06 for the mean CATE prediction (*p* < 0.001), and a coefficient of 0.94 for the OOB prediction (*p* = 0.014), suggesting that the model effectively captured treatment heterogeneity. The selected hyperparameter values were summarized in Supplementary Table S4, and the calibration plot was provided in Supplementary Figure S4.

**Figure 2:**
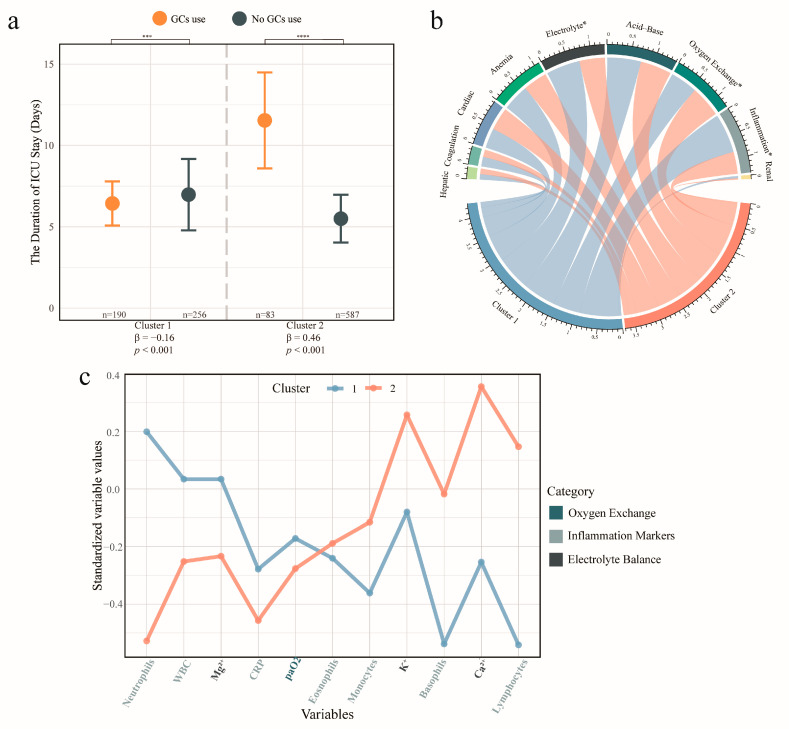
Characteristics of Subclasses in the Whole Cohort. (**a**) The dot plot with error bars comparing the duration of intensive care unit (ICU) stay between glucocorticoids (GC) users and non-users across subclasses in the whole cohort. The top section presents T-test results for the duration of ICU stay between the two subclasses, while the bottom section shows the adjusted β coefficients and *p*-values between GC use and duration of ICU stay from multivariable GLM for each subclass, along with the number of GC users and non-users in each subclass. “***” indicates *p* < 0.001, “****” represents *p* < 0.0001. (**b**) Chord diagram showing the proportion of abnormal laboratory values across different test categories in cluster 1 and 2. Asterisks (*) indicate significant differences in the proportions of abnormalities between the two clusters (*p* < 0.05). (**c**) The line plot depicting the median z-score–standardized values of laboratory tests in Cluster 1 and Cluster 2. The x-axis lists individual laboratory variables, with label colors denoting their corresponding test categories as defined in the right legend, while the y-axis represents the median z-score for each cluster. Only variables from categories with significant between-cluster differences in panel (**b**) are shown. Z-scores were standardized across the entire cohort. Values above 0 indicate levels higher than the cohort average, whereas values below 0 indicate lower-than-average levels. Abbreviations: GCs: Glucocorticoids, ICU: intensive care unit, WBC: white blood cell, Mg^2+^: serum magnesium ion, CRP: C-reactive protein, paO_2_: arterial oxygen partial pressure, K^+^: serum potassium ion, Ca^2+^: serum calcium ion.

As shown in Figure 2a and Table 1, multivariable GLM analysis revealed a significant negative association between GC use and the duration of ICU stay in Cluster 1 (β = −0.16 log-days, *p* < 0.001) and a significant positive association in Cluster 2 (β = 0.46 log-days, *p* < 0.001), indicating that patients in Cluster 1 are more likely to benefit from GC therapy than those in Cluster 2. A similar pattern was identified for GC treatment intensity. Higher initial doses were associated with shorter ICU stays in Cluster 1 (β = −0.14 log-days, *p* < 0.001), but with longer stays in Cluster 2 (β = 0.34 log-days, *p* < 0.001).

As presented in Table 2, patients in Cluster 1 were older than those in Cluster 2 (median [IQR]: 216.00 days [98.00,433.75] vs. 58.00 days [43.00,88.75], *p* < 0.001). They also demonstrated a greater need for MV (46.86% vs. 23.73%, *p* < 0.001), a longer duration of ICU stays (median [IQR]: 6.71 days [5.08,8.79] vs. 5.79 days [4.08,7.92], *p* < 0.001), a greater likelihood to have GC treatment (42.60% vs. 12.39%, *p* < 0.001) and more comorbidities (32.89% vs. 23.88%, *p =* 0.001).

In the laboratory tests, more electrolyte disturbances, and elevated inflammation levels were revealed in Cluster 1 (all *p* < 0.001, Figure 2b and Supplementary Table S5). Conversely, a higher incidence of abnormalities in oxygen exchange was shown in Cluster 2 (*p* = 0.03). These differences were further reflected in the detailed laboratory results (Figure 2c and Supplementary Table S6). Cluster 1 exhibited higher white blood cell (WBC), neutrophil counts, and CRP levels, along with lower counts of lymphocytes (all *p* < 0.001). Whereas Cluster 2 patients presented elevated serum potassium and calcium ion levels, reduced magnesium ion levels, and lower arterial oxygen partial pressure (all *p* < 0.01).

As shown in Table 3, Cluster 2 presented a greater prevalence of viral infections (47.31% vs. 40.81%, *p* = 0.06), including a higher level of Respiratory Syncytial Virus (RSV) infection (42.96% vs. 32.18%, *p =* 0.003). In addition, Cluster 1 had a greater proportion of atypical bacterial infections (8.30% vs. 2.84%, *p* < 0.001). Additionally, bacterial infections were more common in Cluster 1 (43.72% vs. 35.52%, *p =* 0.02), including higher incidences of *Haemophilus influenzae* (14.55% vs. 7.13%, *p =* 0.001) and *Streptococcus pneumoniae* infections (13.56% vs. 8.41%, *p =* 0.03).

### 3.3. Subclass Identification and Intergroup Analysis in the MV Group

The subclass analysis was also conducted for the MV group. The final optimal model also demonstrated good calibration, with a mean CATE prediction coefficient of 1.00 (*p =* 0.04), and an OOB prediction coefficient of 1.25 (*p =* 0.001). The final hyperparameter values are reported in Supplementary Table S4, and calibration results are shown in Supplementary Figure S4.

As shown in Supplementary Figure S5a and Table 1, GLM analysis revealed a significant negative association between GC use and both the duration of ICU stay (β = −0.34 log-days, *p* < 0.001) and MV duration (β = −0.35 log-hours, *p* < 0.001) in Cluster 1. In contrast, a significant positive association was observed in Cluster 2 for the duration of ICU stays (β = 0.36 log-days, *p* < 0.001) and MV duration (β = 0.46 log-hours, *p* < 0.001). Consistent results were obtained for GC treatment intensity. Higher initial doses were linked to shorter ICU stay (β = −0.21 log-days, *p* < 0.001) and MV duration (β = −0.29 log-hours, *p* < 0.001) in Cluster 1, but to longer ICU stay (β = 0.26 log-days, *p* < 0.001) and MV duration (β = 0.30 log-hours, *p* < 0.001) in Cluster 2.

As detailed in Table 4, patients in Cluster 1 were significantly older than those in Cluster 2 (median [IQR]: 240.00 days [104.00,472.50] vs. 85.00 days [51.00,210.00], *p* < 0.001). Additionally, patients in Cluster 1 exhibited a longer duration of MV (median [IQR]: 120.61 h [77.72,180.98] vs. 94.32 h [69.95,154.08], *p* = 0.04), were more likely to receive GC therapy (56.60% vs. 30.62%, *p* < 0.001).

The laboratory test results (Supplementary Figure S5b,c, Supplementary Tables S7 and S8) of the MV group revealed that Cluster 1 had higher inflammation levels, characterized by increased neutrophil counts and elevated CRP levels, along with reduced lymphocyte counts (all *p* < 0.001). In contrast, patients in Cluster 2 demonstrated a greater prevalence of cardiac dysfunction, as evidenced by significantly higher levels of creatine kinase (CK) and creatine kinase- myocardial band (CK-MB). (all *p ≤* 0.001).

In the etiological analysis (Supplementary Table S9), Cluster 2 presented a greater RSV infection rate than Cluster 1 (53.85% vs. 36.50%, *p =* 0.02). Whereas Cluster 1 had higher rates of *Haemophilus influenzae* infection (12.41% vs. 5.49%, *p =* 0.12).

### 3.4. Variable Importance Analysis and Personalized Analysis of Age via SHAP

**Figure 3:**
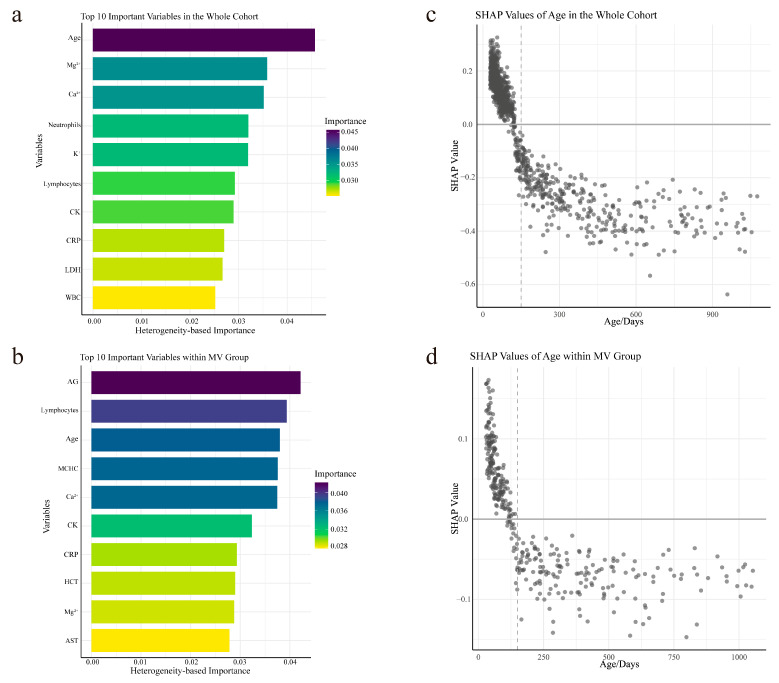
Feature Importances and Age Influence on the Individual Treatment Effects. (**a**,**b**) Top 10 variables contributing to the glucocorticoids (GC) treatment effects, ranked by their importance in the causal forest model. Panel (**a**) shows variables associated with GC effects on ICU length of stay in the whole cohort, while panel (**b**) displays those influencing GC effects on mechanical ventilation duration in the MV subgroup. (**c**,**d**) SHAP (Shapley additive explanations) dependence plots showing how age influences the predicted individual treatment effects (ITEs) in the whole cohort (**c**) and MV subgroup (**d**). Each dot represents a patient, and negative SHAP values indicate greater predicted benefit from GCs. The dashed line marks the 150-day threshold used for stratification. Abbreviations: Mg^2+^: serum magnesium ion, Ca^2+^: serum calcium ion, K^+^: serum potassium ion, CK: creatine kinase, CRP: C-reactive protein, LDH: lactate dehydrogenase, WBC: white blood cell, AG: anion gap, MCHC: mean corpuscular hemoglobin concentration, HCT: hematocrit, AST: aspartate aminotransferase, SHAP: Shapley Additive explanations.

The primary determinants influencing the of GCs’ efficacy on ICU stay duration in the overall cohort included age; serum magnesium concentration, and calcium ion levels (Figure 3a). In contrast, within the MV group, the key factors influencing GCs’ impact on ICU stay duration were identified as the anion gap, lymphocyte count, and age (Figure 3b).

Since age was identified as a key variable in both the whole cohort and the MV group, the SHAP methodology was applied to evaluate its impact on the ITE for each patient. As shown in Figure 3c,d, age was inversely correlated with SHAP values. Patients older than 150 days exhibited negative SHAP values in both the whole cohort and the MV group, indicating that GC therapy was associated with a reduction in ICU stay and a shorter duration of MV in this age group.

With 150 days as the cutoff value for age stratification, Supplementary Table S10 revealed a significantly higher proportion of viral infections in the younger group than in the older age group (46.95% vs. 39.71%, *p =* 0.046). Notably, the rate of RSV infection was significantly greater among younger patients than among older patients (43.81% vs. 27.52%, *p* < 0.001). In contrast, the older group had markedly greater proportions of atypical infections (13.52% vs. 2.64%, *p* < 0.001) and bacterial infections (44.64% vs. 36.19%, *p =* 0.02) than did the younger group.

### 3.5. Sensitivity Analysis

Sensitivity analyses demonstrated that cluster assignments based on both complete-case and missing-indicator approaches showed moderate-to-high agreement with the primary analysis in the whole cohort and the MV subgroup (Supplementary Figure S6). Under both strategies, Cluster 1 and Cluster 2 consistently corresponded to the benefit and non-benefit groups (Supplementary Table S11). The benefit group was further characterized by older age, lower RSV prevalence, and higher inflammatory markers (Supplementary Table S12).

Using the causal forest, the estimated ATE for the whole cohort was 1.64 (days, 95% CI: 1.21–2.06). As a propensity score–based method, the overlap-weighted treatment effect was 1.19 (days, 95% CI: 0.56–1.82). The covariate balance diagnostic for the propensity score method was shown in Supplementary Figure S7. When stratifying patients into four quartiles of predicted treatment effect, a clear gradient of glucocorticoid benefit was observed from Quartile 4 (least benefit) to Quartile 1 (greatest benefit) across both cohorts (Supplementary Table S13). Moreover, Quartile 1 patients, compared with Quartile 4, were consistently older, had lower RSV prevalence, and exhibited higher inflammatory states (Supplementary Table S14).

## 4. Discussion

In this retrospective study of infants and toddlers with severe CAP, we observed significant HTE of GCs and identified a potential subclass of patients who are more likely to respond to GCs, based on causal forest analysis. In both the whole cohort and the MV group, older patients, those with elevated inflammation were more likely to benefit from GCs. Conversely, patients with RSV infection was associated with a lack of clinical benefit, with no significant shortening of ICU stays or MV duration. Variable importance analysis highlighted age and anion gap as key predictors of GC efficacy. SHAP analysis of age indicated that age was positively related to the efficacy of GCs.

Similar HTE of GCs have been observed in adults with CAP. Studies show that GC therapy is effective only in severe cases, where it significantly reduces mortality, with no benefit in non-severe cases [15,16]. A stratified RCT analysis revealed that oral dexamethasone significantly shortened hospital stays only in patients with elevated inflammation markers, with no benefit observed in those with low WBC counts [49]. These outcomes highlight the necessity for further refinement of the indications for GC therapy in adults with CAP.

Our study revealed that in both the whole cohort and the MV group, patients with higher levels of inflammation, characterized by elevated WBC and neutrophil counts alongside reduced lymphocyte counts, benefited more from GC treatment. This finding is consistent with a post hoc analysis of adult CAP [49]. Previous studies have also shown that patients with high neutrophil and low lymphocyte counts tend to have more severe CAP [49], suggesting disease severity may be an indicator of GC efficacy. This hypothesis was confirmed in our subclass analysis of the whole cohort, which demonstrated that infants and toddlers with more severe disease—longer ICU stays, greater need for MV, and more comorbidities—derived greater benefits from GCs [15,16].

In the whole cohort, we also found that patients with bacterial infection were more likely to benefit from GC therapy, whereas those with RSV infection were less likely to benefit from it. Previous meta-analyses in adults with CAP [50] have confirmed that GC therapy can reduce hospital mortality in severe bacterial CAP, with no significant benefit for non-COVID-19 viral pneumonia. Our findings extend this evidence, suggesting that infants and toddlers with bacterial CAP were more likely to be responsive to GC therapy than those with viral CAP. Also, a multicenter RCT on the efficacy of dexamethasone in children mechanically ventilated for RSV-related lower respiratory tract found no significant reduction in ventilation duration [51]. This finding and our conclusions imply that GC therapy may not be suitable for treating pediatric pneumonia caused by RSV.

SHAP analysis revealed a positive correlation between age and GC therapy effectiveness, likely due to differences in etiological distribution by age. Older patients had a higher prevalence of bacterial infections, whereas younger patients presented higher rates of viral infections, especially RSV. As GC therapy is more effective for bacterial infections and less effective for RSV pneumonia, these age-related differences in GC effectiveness may be linked to the underlying variations in pathogen types. However, this finding requires validation in other pediatric age groups, such as neonates and school-aged children, whose immune maturation, host responses, and predominant pathogens differ substantially from those of infants and toddlers [52,53]. Moreover, pathogen distributions in the PICU differ significantly from those in less severe settings. Therefore, whether this age-related trend applies to the settings outside the ICU also needs more validation [54].

Our study has several limitations. First, as our study included only infants and toddlers from a single tertiary PICU, generalizability to older children and to settings with different pathogen distributions remains uncertain, warranting validation in broader populations and contexts. Second, despite using causal forest models and a wide range of covariates to reduce bias, our retrospective study is still limited by unmeasured confounding, particularly from the absence of quantifiable imaging data and certain laboratory markers. Third, our endpoints are short-term and do not include harder outcomes such as mortality, readmissions, or long-term respiratory sequelae, which were either rare or not consistently captured in this retrospective single-centre dataset. Fourth, steroid-related adverse events were incompletely coded, precluding robust comparative safety assessment. Future multi-centre prospective studies with longitudinal follow-up and predefined, adjudicated safety outcomes are required to quantify the benefit–risk balance of glucocorticoids in this population. Finally, although the exploratory, data-driven approach used to define GC responders provides a hypothesis-generating framework, it has inherent limitations, including potential optimism bias, and requires further validation in future studies. And the optimal glucocorticoid regimen for this subgroup remains unclear and warrants further investigation using methods suited for continuous treatments.

## 5. Conclusions

In summary, this study is the first to explore the HTE of GCs among infants and toddlers with severe CAP. Our study revealed that older patients and those with higher inflammation levels were more likely to benefit from GC treatment, with no such benefit observed in those with RSV infections. These insights could guide more personalized treatment strategies for the use of GCs and inform future clinical trials in this population.

## Figures and Tables

**Table 1:** Effect of Glucocorticoids Therapy on Clinical Outcomes Across Subclasses in the Whole Cohort and the Mechanically Ventilated Group.

		Cluster 1				Cluster 2			
Outcomes	Treatment	β(log-time)	95%CI	*p*	Adjusted *p*	β(log-time)	95%CI	*p*	Adjusted *p*
Whole Cohort									
Duration of ICU stay (Days)	GC Use	−0.16	[−0.25,−0.08]	<0.001 *	<0.001 *	0.46	[0.37,0.55]	<0.001 *	<0.001 *
	Duration of GC treatment	−0.01	[−0.02,0.01]	0.50	0.57	0.07	[0.06,0.09]	<0.001 *	<0.001 *
	GC treatment intensity	−0.14	[−0.20,−0.08]	<0.001 *	<0.001 *	0.34	[0.26,0.40]	<0.001 *	<0.001 *
	Time to GC initiation	0.01	[−0.05,0.07]	0.75	0.78	0.01	[−0.02,0.03]	0.64	0.70
Mechanically Ventilated Group									
Duration of ICU stay (Days)	GC Use	−0.34	[−0.46,−0.24]	<0.001 *	<0.001 *	0.36	[0.28,0.46]	<0.001 *	<0.001 *
	Duration of GC treatment	−0.02	[−0.04,−0.004]	0.02 *	0.03 *	0.06	[0.04,0.07]	<0.001 *	<0.001 *
	GC treatment intensity	−0.21	[−0.30,−0.13]	<0.001 *	<0.001 *	0.26	[0.19,0.34]	<0.001 *	<0.001 *
	Time to GC initiation	−0.005	[−0.08,0.07]	0.89	0.89	−0.04	[−0.10,0.01]	0.10	0.13
Mechanical Ventilation Duration (Hours)	GC Use	−0.35	[−0.51,−0.19]	<0.001 *	<0.001 *	0.46	[0.34,0.60]	<0.001 *	<0.001 *
	Duration of GC treatment	−0.01	[−0.041,0.01]	0.33	0.42	0.07	[0.05,0.09]	<0.001 *	<0.001 *
	GC treatment intensity	−0.29	[−0.42,−0.17]	<0.001 *	<0.001 *	0.30	[0.19,0.42]	<0.001 *	<0.001 *
	Time to GC initiation	−0.05	[−0.16,0.06]	0.37	0.44	0.08	[0.03,0.14]	0.006 *	0.009 *

Note: β values are the coefficients estimated from generalized linear models for the corresponding variables. Adjusted *p*-values were corrected for multiple comparisons using the Benjamini–Hochberg procedure. Abbreviations: CI: confidence intervals, ICU: intensive care unit, GC: glucocorticosteroid, * denotes *p* < 0.05.

**Table 2:** Clinical Characteristics Comparison Between Subclasses in the Whole Cohort.

	Overall	Cluster 1	Cluster 2	*p*	Adjusted *p*
Characteristics	1116	446	670		
Age (Median [IQR], Days)	82.50 [51.00,200.00]	216.00 [98.00,433.75]	58.00 [43.00,88.75]	<0.001 *	<0.001 *
Patients Requiring Mechanical Ventilation (%)	368 (32.97)	209 (46.86)	159 (23.73)	<0.001 *	<0.001 *
The duration of ICU stay, Days (median [IQR])	5.96 [4.71,8.24]	6.71 [5.08,8.79]	5.79 [4.08,7.92]	<0.001 *	<0.001 *
Mechanical Ventilation Duration, Hours (median [IQR])	109.10 [69.67,158.93]	109.83 [68.75,140.99]	106.62 [70.21,178.00]	0.27	0.31
Patients Receiving GC Treatment (%)	273 (24.46)	190 (42.60)	83 (12.39)	<0.001 *	<0.001 *
The duration of GC treatment, Days (mean (SD))	5.70 (2.26)	5.48 (1.95)	6.18 (2.79)	0.02 *	0.03 *
GC treatment intensity, mg/(kg·day) (mean (SD))	1.24 (0.45)	1.23 (0.45)	1.28 (0.45)	0.38	0.43
Time to GC initiation, Days (median [IQR])	0.00 [0.00,1.00]	0.00 [0.00,0.00]	0.00 [0.00,1.00]	0.02 *	0.03 *
Comorbidities	310 (27.78)	149 (32.89)	160 (23.88)	<0.001 *	0.001 *
Circulatory System Disease (%)	174 (15.59)	83 (18.61)	91 (13.58)	0.03 *	0.04 *
Blood Disease (%)	62 (5.56)	46 (10.31)	16 (2.39)	<0.001 *	<0.001 *
Respiratory Disease (%)	29 (2.60)	14 (3.14)	15 (2.24)	0.46	0.52
Digestive Disease (%)	63 (5.65)	15 (3.36)	48 (7.16)	0.01 *	0.02 *
Nervous System Disease(%)	38 (3.41)	23 (5.16)	15 (2.24)	0.01 *	0.02 *
Chromosomal Anomalies(%)	17 (1.52)	13 (2.91)	4 (0.60)	0.004 *	0.009 *

Note: Except where indicated, the data are the numbers of patients, with percentages in parentheses. Adjusted *p*-values were corrected for multiple comparisons using the Benjamini–Hochberg procedure. Abbreviations: IQR: Interquartile range, ICU: intensive care unit, GC: glucocorticosteroid, SD: Standard deviation. * denotes *p* < 0.05.

**Table 3:** Etiological Comparison among Subclasses within the Whole Cohort.

	Overall	Cluster 1	Cluster 2	*p*	Adjusted *p*
	1116	446	670		
Viral Infection (%)	499 (44.71)	182 (40.81)	317 (47.31)	0.04 *	0.06
Influenza B (%)	10 (1.06)	3 (0.80)	7 (1.23)	0.75	0.79
Parainfluenza virus (%)	97 (10.28)	36 (9.57)	61 (10.74)	0.64	0.76
Respiratory syncytial virus (%)	365 (38.67)	121 (32.18)	244 (42.96)	0.001 *	0.003 *
Influenza A (%)	24 (2.54)	15 (3.99)	9 (1.58)	0.04 *	0.07
Adenovirus (%)	33 (3.50)	22 (5.85)	11 (1.94)	0.003 *	0.007 *
Atypical Infection (%)	56 (6.20)	37 (8.30)	19 (2.84)	<0.001 *	<0.001 *
*Mycoplasma pneumoniae* (%)	54 (5.98)	37 (8.30)	17 (2.54)	<0.001 *	<0.001 *
*Chlamydophila* *pneumoniae* (%)	2 (0.22)	0 (0.00)	2 (0.30)	0.65	0.76
Bacterial Infection (%)	433 (38.80)	195 (43.72)	238 (35.52)	0.007 *	0.02 *
*Escherichia coli* (%)	38 (3.99)	15 (4.01)	23 (4.03)	1.00	1.00
*Haemophilus influenzae* (%)	96 (10.07)	55 (14.55)	41 (7.13)	<0.001 *	0.001 *
*Klebsiella pneumoniae* (%)	94 (9.86)	44 (11.76)	50 (8.73)	0.16	0.22
*Streptococcus pneumoniae* (%)	99 (10.39)	51 (13.56)	48 (8.41)	0.02 *	0.03 *
*Staphylococcus aureus* (%)	162 (17.00)	52 (13.83)	110 (19.06)	0.044 *	0.07
*Pseudomonas aeruginosa* (%)	19 (2.00)	12 (3.20)	7 (1.23)	0.06	0.09
*Acinetobacter baumannii* (%)	31 (3.25)	15 (4.02)	16 (2.80)	0.40	0.52

Note: Except where indicated, the data are the numbers of patients, with percentages in parentheses. Adjusted *p*-values were corrected for multiple comparisons using the Benjamini–Hochberg procedure. * denotes *p* < 0.05.

**Table 4:** Clinical Characteristics Comparison Between Subclasses in the Mechanical Ventilation Group.

	Overall	Cluster 1	Cluster 2	*p*	Adjusted *p*
Characteristics	368	159	209		
Age, Days (Median [IQR])	127.00 [61.00,338.50]	240.00 [104.00,472.50]	85.00 [51.00,210.00]	<0.001 *	<0.001 *
The duration of ICU stay, Days (median [IQR])	7.04 [5.83,9.97]	7.54 [5.85,10.48]	7.04 [5.83,9.88]	0.60	0.72
Mechanical Ventilation Duration, Hours (median [IQR])	109.35 [70.30,163.43]	120.61 [77.72,180.98]	94.32 [69.95,154.08]	0.02 *	0.04 *
Patients Receiving GC Treatment (%)	154 (41.85)	90 (56.60)	64 (30.62)	<0.001 *	<0.001 *
The duration of GC treatment, Days (mean (SD))	5.86 (2.39)	5.47 (1.89)	6.41 (2.89)	0.02 *	0.04 *
GC treatment intensity, mg/(kg·day) (mean (SD))	1.20 (0.41)	1.19 (0.36)	1.22 (0.49)	0.69	0.78
Time to GC initiation, Days (median [IQR])	0.00 [0.00,1.00]	0.00 [0.00,1.00]	0.00 [0.00,1.00]	0.27	0.37
Comorbidities	132 (35.87)	62 (38.99)	70 (33.49)	0.89	1.00
Circulatory System Disease (%)	74 (20.11)	30 (18.87)	44 (21.05)	0.33	0.62
Blood Disease (%)	39 (10.60)	23 (14.47)	16 (7.66)	0.05	0.35
Respiratory Disease (%)	15 (4.08)	9 (5.66)	6 (2.87)	0.28	0.61
Digestive Disease (%)	11 (2.99)	3 (1.89)	8 (3.83)	0.44	0.64
Nervous System Disease (%)	20 (5.43)	11 (6.92)	9 (4.31)	0.39	0.63
Chromosomal Anomalies (%)	12 (3.26)	5 (3.14)	7 (3.35)	1.00	1.00

Note: Except where indicated, the data are the numbers of patients, with percentages in parentheses. Adjusted *p*-values were corrected for multiple comparisons using the Benjamini–Hochberg procedure. IQR: Interquartile range, ICU: intensive care unit, GC: glucocorticosteroid, SD: Standard deviation. * denotes *p* < 0.05.

## Data Availability

The datasets used and/or analysed during the current study are available from the corresponding author on reasonable request.

## Supplementary Materials

The following supporting information can be downloaded at: https://www.mdpi.com/article/10.3390/biomedicines13102333/s1, Supplementary Methods S1: Diagnostic Criteria, Supplementary Methods S2: Severity Classification Criteria, Supplementary Methods S3: Covariate Selection, Supplementary Methods S4: Classification of Infection Types, Supplementary Methods S5: Classification of Laboratory Tests, Supplementary Methods S6: Definition of the Comorbidities, Supplementary Methods S7: Model Validation; Figure S1: Enrollment Flowchart of the Study; Figure S2: Directed Acyclic Graph for Confounding Control; Figure S3: Diagnostic Plots for the Causal Forest Model; Figure S4: Calibration of the Final Causal Forest Models in the Whole Cohort and MV Subgroup; Figure S5: Characteristics of Subclasses in the Mechanical Ventilation (MV) Group; Figure S6: Sensitivity Analysis of Cluster Assignment under Different Missing Data Approaches; Figure S7: Covariate Balance Before and After Overlap Weighting Based on Logistic Regression Propensity Scores; Table S1: Distribution of Patients by Type of Glucocorticoid Administered; Table S2: Missingness of Candidate Covariates; Table S3: Clinical Characteristics Comparison Between GCs and Non-GCs Users in the Whole Cohort; Table S4: Hyperparameters of the Final Causal Forest Models; Table S5: Comparison of Laboratory Test Abnormalities Between Subclasses in the Whole Cohort; Table S6: Comparison of Laboratory Test Results Between Subclasses in the Whole Cohort; Table S7: Comparison of Laboratory Test Abnormalities Between Subclasses in the Mechanical Ventilation Group; Table S8: Comparison of Laboratory Test Results Between Subclasses in the Mechanical Ventilation Group; Table S9: Etiological Comparison among Subclasses within the Mechanical Ventilation Cohort; Table S10: Age-Stratified Comparison of Etiological Distribution in the Whole Cohort; Table S11: GLMs Validation of Subgroup Effectiveness under Missing-Data Sensitivity Analyses; Table S12: Characteristic Analysis Between Subclasses Based on Missing-Data Sensitivity Analysis; Table S13: Association Between Glucocorticoid Use and Clinical Outcomes by Quartiles of Individual Treatment Effects; Table S14: Characteristic Analysis Across Quartile-Based ITE Subclasses.

## Abbreviations

CAP	Community-acquired pneumonia
GCs	Glucocorticoids
HTE	Heterogeneous treatment effect
ITEs	Individual treatment effects
SHAP	Shapley Additive Explanations
ICU	Intensive care unit
RSV	Respiratory Syncytial Virus

## References

[1] Lee G.E., Lorch S.A., Sheffler-Collins S., Kronman M.P., Shah S.S. (2010). National Hospitalization Trends for Pediatric Pneumonia and Associated Complications. Pediatrics.

[2] Grimwood K., Chang A.B. (2015). Long-Term Effects of Pneumonia in Young Children. Pneumonia.

[3] Burrows B., Knudson R.J., Lebowitz M.D. (1977). The Relationship of Childhood Respiratory Illness to Adult Obstructive Airway Disease. Am. Rev. Respir. Dis..

[4] Stern A., Skalsky K., Avni T., Carrara E., Leibovici L., Paul M. (2017). Corticosteroids for Pneumonia. Cochrane Database Syst. Rev..

[5] Blum C.A., Nigro N., Briel M., Schuetz P., Ullmer E., Suter-Widmer I., Winzeler B., Bingisser R., Elsaesser H., Drozdov D. (2015). Adjunct Prednisone Therapy for Patients with Community-Acquired Pneumonia: A Multicentre, Double-Blind, Randomised, Placebo-Controlled Trial. Lancet.

[6] Meijvis S.C., Hardeman H., Remmelts H.H., Heijligenberg R., Rijkers G.T., van Velzen-Blad H., Voorn G.P., van de Garde E.M., Endeman H., Grutters J.C. (2011). Dexamethasone and Length of Hospital Stay in Patients with Community-Acquired Pneumonia: A Randomised, Double-Blind, Placebo-Controlled Trial. Lancet.

[7] Fernández-Serrano S., Dorca J., Garcia-Vidal C., Fernández-Sabé N., Carratalà J., Fernández-Agüera A., Corominas M., Padrones S., Gudiol F., Manresa F. (2011). Effect of Corticosteroids on the Clinical Course of Community-Acquired Pneumonia: A Randomized Controlled Trial. Crit. Care.

[8] Torres A., Sibila O., Ferrer M., Polverino E., Menendez R., Mensa J., Gabarrús A., Sellarés J., Restrepo M.I., Anzueto A. (2015). Effect of Corticosteroids on Treatment Failure Among Hospitalized Patients With Severe Community-Acquired Pneumonia and High Inflammatory Response: A Randomized Clinical Trial. JAMA.

[9] Confalonieri M., Urbino R., Potena A., Piattella M., Parigi P., Puccio G., Della Porta R., Giorgio C., Blasi F., Umberger R. (2005). Hydrocortisone Infusion for Severe Community-Acquired Pneumonia. Am. J. Respir. Crit. Care Med..

[10] Dequin P.F., Meziani F., Quenot J.P., Kamel T., Ricard J.D., Badie J., Reignier J., Heming N., Plantefève G., Souweine B. (2023). Hydrocortisone in Severe Community-Acquired Pneumonia. N. Engl. J. Med..

[11] Wittermans E., Vestjens S.M.T., Spoorenberg S.M.C., Blok W.L., Grutters J.C., Janssen R., Rijkers G.T., Smeenk F.W.J.M., Voorn G.P., van de Garde E.M.W. (2021). Adjunctive Treatment with Oral Dexamethasone in Non-ICU Patients Hospitalised with Community-Acquired Pneumonia: A Randomised Clinical Trial. Eur. Respir. J..

[12] Meduri G.U., Shih M.-C., Bridges L., Martin T.J., El-Solh A., Seam N., Davis-Karim A., Umberger R., Anzueto A., Sriram P. (2022). Low-Dose Methylprednisolone Treatment in Critically Ill Patients with Severe Community-Acquired Pneumonia. Intensive Care Med..

[13] Snijders D., Daniels J.M.A., de Graaff C.S., van der Werf T.S., Boersma W.G. (2010). Efficacy of Corticosteroids in Community-Acquired Pneumonia. Am. J. Respir. Crit. Care Med..

[14] Saleem N., Kulkarni A., Snow T.A.C., Ambler G., Singer M., Arulkumaran N. (2023). Effect of Corticosteroids on Mortality and Clinical Cure in Community-Acquired Pneumonia: A Systematic Review, Meta-Analysis, and Meta-Regression of Randomized Control Trials. Chest.

[15] Pitre T., Abdali D., Chaudhuri D., Pastores S.M., Nei A.M., Annane D., Rochwerg B., Zeraatkar D. (2023). Corticosteroids in Community-Acquired Bacterial Pneumonia: A Systematic Review, Pairwise and Dose-Response Meta-Analysis. J. Gen. Intern. Med..

[16] Horby P., Lim W.S., Emberson J.R., Mafham M., Bell J.L., Linsell L., Staplin N., Brightling C., Ustianowski A., RECOVERY Collaborative Group (2021). Dexamethasone in Hospitalized Patients with COVID-19. N. Engl. J. Med..

[17] An J., Baek K.S., Lee S. (2022). The Effects of Systemic Corticosteroid on Pediatric Community-Acquired Pneumonia: Comprehensive Review. Life Cycle.

[18] Ozsurekci Y., Aykac K., Demir O.O., Ilbay S., Kesici S., Karakaya J., Cengiz A.B. (2023). Methylprednisolone Use in Children with Severe Pneumonia Caused by Severe Acute Respiratory Syndrome Coronavirus 2. Pediatr. Int..

[19] Zhang L., Wang L., Xu S., Li H., Chu C., Liu Q., Zhou J., Zhang W., Huang L. (2020). Low-Dose Corticosteroid Treatment in Children With Mycoplasma Pneumoniae Pneumonia: A Retrospective Cohort Study. Front. Pediatr..

[20] Nagy B., Gaspar I., Papp A., Bene Z., Nagy B., Voko Z., Balla G. (2013). Efficacy of Methylprednisolone in Children with Severe Community Acquired Pneumonia. Pediatr. Pulmonol..

[21] Kim H.S., Sol I.S., Li D., Choi M., Choi Y.J., Lee K.S., Seo J.H., Lee Y.J., Yang H.-J., Kim H.H. (2019). Efficacy of Glucocorticoids for the Treatment of Macrolide Refractory Mycoplasma Pneumonia in Children: Meta-Analysis of Randomized Controlled Trials. BMC Pulm. Med..

[22] Luo Z., Luo J., Liu E., Xu X., Liu Y., Zeng F., Li S., Fu Z. (2014). Effects of Prednisolone on Refractory Mycoplasma Pneumoniae Pneumonia in Children. Pediatr. Pulmonol..

[23] Han J.Y., Yang E.A., Rhim J.-W., Han S.B. (2021). Effects of Antiviral Therapy and Glucocorticoid Therapy on Fever Duration in Pediatric Patients with Influenza. Medicina.

[24] Ambroggio L., Test M., Metlay J.P., Graf T.R., Blosky M.A., Macaluso M., Shah S.S. (2015). Adjunct Systemic Corticosteroid Therapy in Children With Community-Acquired Pneumonia in the Outpatient Setting. J. Pediatric Infect. Dis. Soc..

[25] Weiss A.K., Hall M., Lee G.E., Kronman M.P., Sheffler-Collins S., Shah S.S. (2011). Adjunct Corticosteroids in Children Hospitalized with Community-Acquired Pneumonia. Pediatrics.

[26] Tagarro A., Otheo E., Baquero-Artigao F., Navarro M.-L., Velasco R., Ruiz M., Penín M., Moreno D., Rojo P., Madero R. (2017). Dexamethasone for Parapneumonic Pleural Effusion: A Randomized, Double-Blind, Clinical Trial. J. Pediatr..

[27] Yao T.-C., Wang J.-Y., Chang S.-M., Chang Y.-C., Tsai Y.-F., Wu A.C., Huang J.-L., Tsai H.-J. (2021). Association of Oral Corticosteroid Bursts With Severe Adverse Events in Children. JAMA Pediatr..

[28] Varadhan R., Seeger J.D. (2013). Estimation and Reporting of Heterogeneity of Treatment Effects. Developing a Protocol for Observational Comparative Effectiveness Research: A User’s Guide.

[29] Sinha P., Spicer A., Delucchi K.L., McAuley D.F., Calfee C.S., Churpek M.M. (2021). Comparison of Machine Learning Clustering Algorithms for Detecting Heterogeneity of Treatment Effect in Acute Respiratory Distress Syndrome: A Secondary Analysis of Three Randomised Controlled Trials. eBioMedicine.

[30] Wager S., Athey S. (2018). Estimation and Inference of Heterogeneous Treatment Effects Using Random Forests. J. Am. Stat. Assoc..

[31] Athey S., Imbens G. (2016). Recursive Partitioning for Heterogeneous Causal Effects. Proc. Natl. Acad. Sci. USA.

[32] Shiba K., Daoud A., Kino S., Nishi D., Kondo K., Kawachi I. (2022). Uncovering Heterogeneous Associations of Disaster-Related Traumatic Experiences with Subsequent Mental Health Problems: A Machine Learning Approach. Psychiatry Clin. Neurosci..

[33] Osawa I., Goto T., Kudo D., Hayakawa M., Yamakawa K., Kushimoto S., Foster D.M., Kellum J.A., Doi K. (2023). Targeted Therapy Using Polymyxin B Hemadsorption in Patients with Sepsis: A Post-Hoc Analysis of the JSEPTIC-DIC Study and the EUPHRATES Trial. Crit. Care.

[34] Zhou Z., Jian B., Chen X., Liu M., Zhang S., Fu G., Li G., Liang M., Tian T., Wu Z. (2023). Heterogeneous Treatment Effects of Coronary Artery Bypass Grafting in Ischemic Cardiomyopathy: A Machine Learning Causal Forest Analysis. J. Thorac. Cardiovasc. Surg..

[35] You C., Shen Y., Sun S., Zhou J., Li J., Su G., Michalopoulou E., Peng W., Gu Y., Guo W. (2023). Artificial Intelligence in Breast Imaging: Current Situation and Clinical Challenges. Exploration.

[36] Edward J.A., Josey K., Bahn G., Caplan L., Reusch J.E.B., Reaven P., Ghosh D., Raghavan S. (2022). Heterogeneous Treatment Effects of Intensive Glycemic Control on Major Adverse Cardiovascular Events in the ACCORD and VADT Trials: A Machine-Learning Analysis. Cardiovasc. Diabetol..

[37] Goligher E.C., Lawler P.R., Jensen T.P., Talisa V., Berry L.R., Lorenzi E., McVerry B.J., Chang C.-C.H., Leifer E., Bradbury C. (2023). Heterogeneous Treatment Effects of Therapeutic-Dose Heparin in Patients Hospitalized for COVID-19. JAMA.

[38] National Health Commission of the People’s Republic of China (2019). State Administration of Traditional Chinese Medicine Guideline for Diagnosis and Treatment of Community-Acquired Pneumonia in Children (2019 Version). Chin. J. Clin. Infect. Dis..

[39] National Health Commission of the People’s Republic of China Reference Intervals of Blood Cell Analysis for Children. http://www.nhc.gov.cn/wjw/s9492/202105/a85d8b64e0384c98aed8f3157860ee44/files/1739781618961_15816.pdf.

[40] National Health Commission of the People’s Republic of China Reference Intervals of Clinical Biochemistry Tests Commonly Used for Children. http://www.nhc.gov.cn/wjw/s9492/202105/3d5159ef7619452b9842ea9520189a11/files/1739781620270_69076.pdf.

[41] Liu P., Li S., Zheng T., Wu J., Fan Y., Liu X., Gong W., Xie H., Liu J., Li Y. (2023). Subphenotyping Heterogeneous Patients with Chronic Critical Illness to Guide Individualised Fluid Balance Treatment Using Machine Learning: A Retrospective Cohort Study. eClinicalMedicine.

[42] Tibshirani J., Athey S., Friedberg R., Hadad V., Hirshberg D., Miner L., Sverdrup E., Wager S., Wright M. (2024). Grf: Generalized Random Forests.

[43] Athey S., Wager S. (2019). Estimating Treatment Effects with Causal Forests: An Application. Obs. Stud..

[44] Cavanaugh J.E., Neath A.A. (2019). The Akaike Information Criterion: Background, Derivation, Properties, Application, Interpretation, and Refinements. WIREs Comput. Stat..

[45] Lundberg S.M., Lee S.-I. (2017). A Unified Approach to Interpreting Model Predictions. Proceedings of the 31st International Conference on Neural Information Processing Systems.

[46] Lundberg S.M., Nair B., Vavilala M.S., Horibe M., Eisses M.J., Adams T., Liston D.E., Low D.K.-W., Newman S.-F., Kim J. (2018). Explainable Machine-Learning Predictions for the Prevention of Hypoxaemia during Surgery. Nat. Biomed. Eng..

[47] Molnar C., Casalicchio G., Bischl B. (2018). Iml: An R Package for Interpretable Machine Learning. J. Open Source Softw..

[48] Xu Y., Yadlowsky S. Calibration Error for Heterogeneous Treatment Effects. Proceedings of the International Conference on Artificial Intelligence and Statistics.

[49] Wittermans E., van de Garde E.M., Voorn G.P., Aldenkamp A.F., Janssen R., Grutters J.C., Bos W.J.W. (2022). Neutrophil Count, Lymphocyte Count and Neutrophil-to-Lymphocyte Ratio in Relation to Response to Adjunctive Dexamethasone Treatment in Community-Acquired Pneumonia. Eur. J. Intern. Med..

[50] Pirracchio R., Venkatesh B., Legrand M. (2024). Low-Dose Corticosteroids for Critically Ill Adults With Severe Pulmonary Infections: A Review. JAMA.

[51] van Woensel J.B.M., Vyas H., on behalf of the STAR Trial Group (2011). Dexamethasone in Children Mechanically Ventilated for Lower Respiratory Tract Infection Caused by Respiratory Syncytial Virus: A Randomized Controlled Trial. Crit. Care Med..

[52] Liu Y.-N., Zhang Y.-F., Xu Q., Qiu Y., Lu Q.-B., Wang T., Zhang X.-A., Lin S.-H., Lv C.-L., Jiang B.-G. (2023). Infection and Co-Infection Patterns of Community-Acquired Pneumonia in Patients of Different Ages in China from 2009 to 2020: A National Surveillance Study. Lancet Microbe.

[53] Simon A.K., Hollander G.A., McMichael A. (2015). Evolution of the Immune System in Humans from Infancy to Old Age. Proc. Biol. Sci..

[54] Cillóniz C., Ewig S., Polverino E., Marcos M.A., Esquinas C., Gabarrús A., Mensa J., Torres A. (2011). Microbial Aetiology of Community-Acquired Pneumonia and Its Relation to Severity. Thorax.

