# Supplementary material for: Assessing the Heterogeneous Treatment Effects of Glucocorticoids in Infants and Toddlers with Severe Pneumonia
Source: Biomedicines. 2025 Sep 24;13(10):2333. doi: 10.3390/biomedicines13102333 (PMC12561364; doi:10.3390/biomedicines13102333)
Supplement: Supplementary file 1 [file biomedicines-13-02333-s001.zip › biomedicines-3765083-supplementary.pdf]

Supplementary Materials

1. Supplementary Methods 1

1.1. Supplementary Methods S1: Diagnostic Criteria 1

1.2. Supplementary Methods S2: Severity Classification Criteria 1

1.3. Supplementary Methods S3: Covariate Selection 1

1.4. Supplementary Methods S4: Classification of Infection Types 1

1.5. Supplementary Methods S5: Classification of Laboratory Tests 2

1.6. Supplementary Methods S6: Definition of the Comorbidities 2

1.7. Supplementary Methods S7: Model Validation 2

1.8. Reference 3

2. Supplementary Tables 5

2.1. Supplementary Table S1. Distribution of Patients by Type of Glucocorticoid Administered 5

2.2. Supplementary Table S2. Missingness of candidate covariates 5

2.3. Supplementary Table S3. Clinical Characteristics Comparison Between GCs and Non-GCs Users in the Whole Cohort 6

2.4. Supplementary Table S4. Hyperparameters of the Final Causal Forest Models........ 7

2.5. Supplementary Table S5. Comparison of Laboratory Test Abnormalities Between Subclasses in the Whole Cohort. 8

2.6. Supplementary Table S6. Comparison of Laboratory Test Results Between Subclasses in the Whole Cohort. 9

2.7. Supplementary Table S7. Comparison of Laboratory Test Abnormalities Between Subclasses in the Mechanical Ventilation Group. 13

2.8. Supplementary Table S8. Comparison of Laboratory Test Results Between Subclasses in the Mechanical Ventilation Group. 14

2.9. Supplementary Table S9. Etiological Comparison among Subclasses within the Mechanical Ventilation Cohort. 17

2.10. Supplementary Table S10. Age-Stratified Comparison of Etiological Distribution in the Whole Cohort. 18

2.11. Supplementary Table S11. Generalized Linear Models (GLMs) validation of subgroup effectiveness under missing-data sensitivity analyses 19

2.12. Supplementary Table S12. Characteristic analysis between suclasses based on missing-data sensitivity analysis. 20

2.13. Supplementary Table S13. Association between Glucocorticoids Use and Clinical Outcomes by Quartiles of Individual Treatment Effects. 22

2.14. Supplementary Table S14. Characteristic analysis across quartile-based ITE suclasses. 23

3. Supplementary Figures 24

3.1. Supplementary Figure S1. Enrollment flowchart of the study. 24

3.2. Supplementary Figure S2. Directed Acyclic Graph for Confounding Control. 25

3.3. Supplementary Figure S3. Diagnostic Plots for the Causal Forest Model. 25

3.4. Supplementary Figure S4. Calibration of the final causal forest models in the whole cohort and MV subgroup. 26

3.5. Supplementary Figure S5. Characteristics of Subclasses in the Mechanical Ventilation (MV) Group. 27

3.6. Supplementary Figure S6. Sensitivity analysis of cluster assignment under different missing data approaches. 28

3.7. Supplementary Figure S7. Covariate balance before and after overlap weighting based on logistic regression propensity scores. 29

1. Supplementary Methods

1.1. Supplementary Methods S1: Diagnostic Criteria

1) Diagnostic Criteria
   a) Onset in the community.
   b) Clinical manifestations of pneumonia, including:
      i. New onset of cough, sputum production, or exacerbation of preexisting respiratory symptoms, with or without purulent sputum, chest pain, dyspnea, or hemoptysis,
      ii. Fever,
      iii. Signs of lung consolidation and/or auscultation of wet crackles,
      iv. Peripheral white blood cell count (WBC) > 10×10^9/L or < 4×10^9/L, with or without a left shift of neutrophil nuclei.
   c) Chest imaging: showing newly developed patchy infiltrates, lobar/segmental consolidation, ground-glass opacity, or interstitial changes, with or without pleural effusion.
2) A diagnosis of CAP can be established if criteria a and c, along with any one of the items in criteria b, are met, after ruling out other diseases. [1]

1.2. Supplementary Methods S2: Severity Classification Criteria

1) Severe cases are defined by meeting any of the following criteria:
   a) poor general condition,
   b) altered consciousness,
   c) presence of cyanosis, rapid breathing (Respiratory Rate [RR] ≥ 70 breaths/min in infants, RR ≥ 50 breaths/min in children over 1 year), or signs of respiratory distress (moaning, nasal flaring, intercostal/subcostal retractions), intermittent apnea, or oxygen saturation < 92%,
   d) presence of hyperpyrexia or persistent high fever lasting more than 5 days,
   e) signs of dehydration or refusal to eat,
   f) chest X-ray or CT scan findings showing ≥ 2/3 of unilateral lung involvement, multilobe infiltration, pleural effusion, pneumothorax, atelectasis, lung necrosis, or lung abscess,
   g) or occurrence of extrapulmonary complications.
2) All other cases not meeting these criteria are considered mild. [1]

1.3. Supplementary Methods S3: Covariate Selection

To estimate the causal effect of Glucocorticoids (GCs) on pediatric severe pneumonia, we established a conceptual framework to identify a sufficient set of covariates for confounding adjustment. This framework is visually represented by a Directed Acyclic Graph (DAG) in Figure S1. Our objective was to identify and control for baseline variables that are common causes of both treatment selection and the outcome, thereby making the core assumption of conditional exchangeability plausible.

The covariates included in the causal forest model were selected through a two-stage process based on a review of the existing literature in pneumonia and data availability within our dataset. The final adjustment set included a set of baseline demographic and clinical covariates, including age, comorbidities, measures of liver and renal function, inflammatory markers, hematologic parameters, and blood gas values reflecting gas exchange and acid-base balance, which were selected based on their established roles as confounders in previous studies [4–16].

1.4. Supplementary Methods S4: Classification of Infection Types

For each type of infection, the following pathogen tests were performed. A positive result in any test within a category indicates the corresponding bacterial, viral, or atypical pathogen infection.

1) **Bacterial Testing**

   a) Blood bacterial cultures,

   b) Sputum smear and culture: Semi-quantitative sputum culture was performed, and the presence of dominant bacteria was considered pathogenic,

   c) Bronchoalveolar lavage fluid (BALF) bacterial culture and metagenomic next-generation sequencing (mNGS).

2) **Viral Testing: including RSV, parainfluenza virus, influenza virus, and Adenovirus.**

   a) Nasal secretion viral antigen detection: Immunofluorescence was used to detect viral antigens,

   b) Nasal secretion viral nucleic acid detection: Polymerase chain reaction (PCR) was used to detect viral DNA or RNA,

   c) Serum specific antibodies: Specific IgM antibodies were measured.

3) **Atypical Pathogen Testing: including Mycoplasma, Chlamydia.**

   a) Serum specific antibodies: Specific IgG and IgM antibodies were measured.

   b) Nucleic acid detection in nasal secretions or BALF: DNA or RNA corresponding to the pathogen was measured. [1]

1.5. Supplementary Methods S5: Classification of Laboratory Tests

Renal abnormalities were defined as elevated blood urea nitrogen and creatinine. Hepatic abnormalities included elevated alanine aminotransferase and aspartate aminotransferase. Cardiac abnormalities were identified by elevated creatine kinase or creatine kinase myocardial band. Electrolyte abnormalities included deviations in plasma chloride, calcium, sodium, potassium, or magnesium levels. Inflammation was defined as elevated C-reactive protein, white blood cell count, neutrophils, or reduced lymphocytes. Anemia was indicated by reduced red blood cell count, hemoglobin, or hematocrit levels. Coagulation abnormalities were defined as reduced platelets, low platelet ratio, mean platelet volume, or plateletcrit. Acid–base abnormalities included deviations in arterial blood pH, lactate, partial pressure of carbon dioxide, actual base excess, bicarbonate, or anion gap. Oxygen exchange abnormalities included reduced arterial oxygen partial pressure or arterial oxygen saturation. The normal reference ranges for the pediatric laboratory values listed above were defined according to two industry standards [2,3].

1.6. Supplementary Methods S6: Definition of the Comorbidities

According to ICD-9, comorbidities were classified into the following categories: blood diseases (ICD codes 280.0–289.9), nervous system diseases (ICD codes 320–359), circulatory system diseases (ICD codes 390–459), respiratory diseases (ICD codes 460–519, excluding chronic lung disease and aspiration pneumonia), digestive diseases (ICD codes 520–579), and chromosomal anomalies (ICD codes 758–758).

1.7. Supplementary Methods S7: Model Validation

1) Causal forest model Validation

This study validates the causal forest model using two methods: the Calibration Test and the Calibration Plot.

The calibration test follows a best-linear-predictor framework that examines whether variation in the model's predicted individual effects corresponds to genuine variation in outcomes after removing main effects and treatment propensity. Operationally, residualized outcomes are regressed on residualized treatment terms decomposed into (i) an average-effect component and (ii) a differential component that captures heterogeneity. The first coefficient, mean prediction, reflects the model's

estimate of the average treatment effect (ATE); values approaching one indicate accurate calibration of the overall effect. The second, differential prediction, assesses treatment effect heterogeneity: a statistically significant and positive value suggests that the model captures meaningful variation, while a value close to one indicates that the predicted individual effects are correctly scaled relative to the true heterogeneity. To mitigate overfitting, all predictions used in the regression are obtained out-of-bag [21–23].

The calibration plot provides a visual method to assess how well a model's predicted treatment effects align with empirically estimated effects. To construct the plot, patients are first partitioned into five quintiles based on their individual predicted treatment effects. For each quintile, a group-specific average effect was calculated [20]. These empirical ATEs are subsequently plotted against the mean predicted effects for each corresponding group. The plot is interpreted by assessing the alignment of these points with the 45-degree identity line: points falling close to the line indicate good calibration, whereas a slope flatter or steeper than the line suggests the model is underestimating or overestimating heterogeneity, respectively. Given that the calibration test and plot are based on distinct statistical approaches, an individual-level regression versus a group-level estimation, their results may differ. Consequently, we report both diagnostics to offer a complementary perspective on model calibration.

1.8. Reference

1. National Health Commission of the People's Republic of China; State Administration of Traditional Chinese Medicine Guideline for Diagnosis and Treatment of Community-Acquired Pneumonia in Children (2019 Version). *Chin J Clin Infect Dis* **2019**, *12*, 6–13, doi:10.3760/cma.j.issn.1674-2397.2019.01.002.
2. National Health Commission of the People's Republic of China Reference Intervals of Blood Cell Analysis for Children Available online: http://www.nhc.gov.cn/wjw/s9492/202105/19e3455a5dfe47e7a84756dd3968e475.shtml (accessed on 15 September 2024).
3. National Health Commission of the People's Republic of China Reference Intervals of Clinical Biochemistry Tests Commonly Used for Children Available online: http://www.nhc.gov.cn/wjw/s9492/202105/170ca00246014d18b82a61cabf9fdb2f.shtml (accessed on 15 September 2024).
4. An, J.; Baek, K.S.; Lee, S. The Effects of Systemic Corticosteroid on Pediatric Community-Acquired Pneumonia: Comprehensive Review. *Life Cycle* **2022**, *2*, e12, doi:10.54724/lc.2022.e12.
5. Ozsurekci, Y.; Aykac, K.; Demir, O.O.; Ilbay, S.; Kesici, S.; Karakaya, J.; Cengiz, A.B. Methylprednisolone Use in Children with Severe Pneumonia Caused by Severe Acute Respiratory Syndrome Coronavirus 2. *Pediatr Int* **2023**, *65*, e15603, doi:10.1111/ped.15603.
6. Zhang, L.; Wang, L.; Xu, S.; Li, H.; Chu, C.; Liu, Q.; Zhou, J.; Zhang, W.; Huang, L. Low-Dose Corticosteroid Treatment in Children With Mycoplasma Pneumoniae Pneumonia: A Retrospective Cohort Study. *Front Pediatr* **2020**, *8*, 566371, doi:10.3389/fped.2020.566371.
7. Nagy, B.; Gaspar, I.; Papp, A.; Bene, Z.; Nagy Jr, B.; Voko, Z.; Balla, G. Efficacy of Methylprednisolone in Children with Severe Community Acquired Pneumonia. *Pediatr Pulmonol* **2013**, *48*, 168–175, doi:10.1002/ppul.22574.
8. Kim, H.S.; Sol, I.S.; Li, D.; Choi, M.; Choi, Y.J.; Lee, K.S.; Seo, J.H.; Lee, Y.J.; Yang, H.-J.; Kim, H.H. Efficacy of Glucocorticoids for the Treatment of Macrolide Refractory Mycoplasma Pneumonia in Children: Meta-Analysis of Randomized Controlled Trials. *BMC Pulm Med* **2019**, *19*, 251, doi:10.1186/s12890-019-0990-8.

9. Luo, Z.; Luo, J.; Liu, E.; Xu, X.; Liu, Y.; Zeng, F.; Li, S.; Fu, Z. Effects of Prednisolone on Refractory Mycoplasma Pneumoniae Pneumonia in Children. *Pediatr Pulmonol* **2014**, *49*, 377–380, doi:10.1002/ppul.22752.
10. Han, J.Y.; Yang, E.A.; Rhim, J.-W.; Han, S.B. Effects of Antiviral Therapy and Glucocorticoid Therapy on Fever Duration in Pediatric Patients with Influenza. *Medicina* **2021**, *57*, 1385, doi:10.3390/medicina57121385.
11. Ambroggio, L.; Test, M.; Metlay, J.P.; Graf, T.R.; Blosky, M.A.; Macaluso, M.; Shah, S.S. Adjunct Systemic Corticosteroid Therapy in Children With Community-Acquired Pneumonia in the Outpatient Setting. *J Pediatric Infect Dis Soc* **2015**, *4*, 21–27, doi:10.1093/jpids/piu017.
12. Weiss, A.K.; Hall, M.; Lee, G.E.; Kronman, M.P.; Sheffler-Collins, S.; Shah, S.S. Adjunct Corticosteroids in Children Hospitalized with Community-Acquired Pneumonia. *Pediatrics* **2011**, *127*, e255-263, doi:10.1542/peds.2010-0983.
13. Tagarro, A.; Otheo, E.; Baquero-Artigao, F.; Navarro, M.-L.; Velasco, R.; Ruiz, M.; Penín, M.; Moreno, D.; Rojo, P.; Madero, R.; et al. Dexamethasone for Parapneumonic Pleural Effusion: A Randomized, Double-Blind, Clinical Trial. *J Pediatr* **2017**, *185*, 117-123.e6, doi:10.1016/j.jpeds.2017.02.043.
14. Blum, C.A.; Nigro, N.; Briel, M.; Schuetz, P.; Ullmer, E.; Suter-Widmer, I.; Winzeler, B.; Bingisser, R.; Elsaesser, H.; Drozdov, D.; et al. Adjunct Prednisone Therapy for Patients with Community-Acquired Pneumonia: A Multicentre, Double-Blind, Randomised, Placebo-Controlled Trial. *The Lancet* **2015**, *385*, 1511–1518, doi:10.1016/S0140-6736(14)62447-8.
15. Fernández-Serrano, S.; Dorca, J.; Garcia-Vidal, C.; Fernández-Sabé, N.; Carratalà, J.; Fernández-Agüera, A.; Corominas, M.; Padrones, S.; Gudiol, F.; Manresa, F. Effect of Corticosteroids on the Clinical Course of Community-Acquired Pneumonia: A Randomized Controlled Trial. *Critical Care* **2011**, *15*, R96, doi:10.1186/cc10103.
16. Dequin Pierre-François; Meziani Ferhat; Quenot Jean-Pierre; Kamel Toufik; Ricard Jean-Damien; Badie Julio; Reignier Jean; Heming Nicholas; Plantefève Gaëtan; Souweine Bertrand; et al. Hydrocortisone in Severe Community-Acquired Pneumonia. *N Engl J Med* **2023**, *388*, 1931–1941, doi:10.1056/NEJMoa2215145.
17. Wager, S.; Athey, S. Estimation and Inference of Heterogeneous Treatment Effects Using Random Forests. *J Am Stat Assoc* **2018**, *113*, 1228–1242, doi:10.1080/01621459.2017.1319839.
18. Athey, S.; Imbens, G. Recursive Partitioning for Heterogeneous Causal Effects. *Proc Natl Acad Sci U S A* **2016**, *113*, 7353–7360, doi:10.1073/pnas.1510489113.
19. Athey, S.; Wager, S. Estimating Treatment Effects with Causal Forests: An Application 2019.
20. Xu, Y.; Yadlowsky, S. Calibration Error for Heterogeneous Treatment Effects. In Proceedings of the International Conference on Artificial Intelligence and Statistics; PMLR, 2022; pp. 9280–9303.

2. Supplementary Tables

2.1. Supplementary Table S1. Distribution of Patients by Type of Glucocorticoid Administered

Type of Glucocorticoids	Patients with any systemic use
**Methylprednisolone**	270
**Dexamethasone**	6
**Prednisone**	6
**Hydrocortisone**	1
**Hydrocortisone-only**	0

Note: Counts indicate the number of patients who received each glucocorticoid type.

2.2. Supplementary Table S2. Missingness of candidate covariates

Covariates	Number of missing values	Percentage missing (%)
**Total lymphocyte**	668	59.86
**B-lymphocyte**	668	59.86
**T-lymphocytes**	668	59.86
**$CD4^+$ T-cells**	668	59.86
**CD8+ T-cells**	668	59.86
**CD4/CD8 Ratio**	668	59.86
**Natural Killer cells**	668	59.86
**Immunoglobulin A**	694	62.19
**Immunoglobulin M**	694	62.19
**Immunoglobulin G**	694	62.19
**Procalcitonin**	471	42.20
**Thrombin Time**	564	50.54
**D-dimer**	564	50.54
**Fibrinogen Degradation Products**	564	50.54
**Prothrombin Time**	564	50.54
**International Normalized Ratio**	564	50.54
**Activated Partial Thromboplastin Time**	564	50.54
**Fibrinogen**	564	50.54
**Heart Rate**	559	50.09
**Respiratory Rate**	559	50.09
**Peripheral Oxygen Saturation**	559	50.09

Note: These covariates were excluded from the main causal forest analysis because more than 40% of values were missing. Percentages are calculated relative to the whole cohort.

2.3. Supplementary Table S3. Clinical Characteristics Comparison Between GCs and Non-GCs Users in the Whole Cohort

	**Overall**	**No GCs use**	**GCs use**	***p***	**Adjusted *p***
**Characteristics**	1116	843	273		
**Age, Days (Median [IQR])**	82.50 [51.00,200.00]	69.00 [48.00, 122.00]	247.00 [92.00, 511.00]	<0.001*	<0.001*
**Patients Requiring Mechanical Ventilation (%)**	368 (32.97)	214 (25.39)	154 (56.41)	<0.001*	<0.001*
**Duration of treatment**					
**The duration of ICU stay, Days (median [IQR])**	5.96 [4.71,8.24]	5.83 [4.50, 7.92]	6.96 [5.71, 9.75]	<0.001*	<0.001*
**Mechanical Ventilation Duration, Hours (median [IQR])**	109.10 [69.67,158.93]	95.82 [67.01, 158.70]	114.48 [79.87, 159.21]	0.10	0.13
**Comorbidities**	310 (27.78)	226 (26.81)	84 (30.77)	0.23	0.37
**Circulatory System Disease (%)**	174 (15.59)	113 (15.01)	61 (16.80)	0.71	0.81
**Blood Disease (%)**	62 (5.56)	33 (3.91)	29 (10.62)	<0.001*	<0.001*
**Respiratory Disease (%)**	29 (2.60)	24 (2.85)	5 (1.83)	0.49	0.65
**Digestive Disease (%)**	63 (5.65)	56 (6.64)	7 (2.56)	0.02*	0.045*
**Nervous System Disease (%)**	38 (3.41)	29 (3.44)	9 (3.30)	1.00	1.00
**Chromosomal Anomalies (%)**	17 (1.52)	16 (1.90)	1 (0.37)	0.13	0.26

Note: Except where indicated, the data are the numbers of patients, with percentages in parentheses. Adjusted *p*-values were corrected for multiple comparisons using the Benjamini–Hochberg procedure. Abbreviations: IQR: Interquartile range, ICU: intensive care unit, GCs: glucocorticosteroids.

2.4. Supplementary Table S4. Hyperparameters of the Final Causal Forest Models.

Hyperparameter	Model for the Whole Cohort	Model for the MV Group
**Sample splitting fraction**	0.48	0.43
**Number of variables tried at each split**	6	2
**Minimum number of samples in a leaf node**	1	1
**Fraction of data used for honest splitting**	0.78	0.64
**Whether to prune leaves after honesty splitting**	0	1
**Splitting regularization parameter**	0.09	0.12
**Penalty for covariate imbalance in splits**	0.38	0.87

Note: This table lists the selected hyperparameters for the final causal forest models in the whole cohort and the mechanically ventilated (MV) subgroup. Tuning was performed via tenfold cross-validation to minimize out-of-bag (OOB) error.

2.5. Supplementary Table S5. Comparison of Laboratory Test Abnormalities Between Subclasses in the Whole Cohort.

	Overall	Cluster 1	Cluster 2	*p*	Adjusted *p*
	1116	446	670		
**Renal Abnormality (%)**	34 (3.64)	18 (4.93)	16 (2.82)	0.13	0.16
**Hepatic Abnormality (%)**	111 (11.88)	43 (11.78)	68 (11.95)	1.00	1.00
**Coagulation Abnormality (%)**	169 (17.77)	79 (20.79)	90 (15.76)	0.06	0.12
**Cardiac Abnormality (%)**	436 (46.83)	157 (43.13)	279 (49.21)	0.08	0.12
**Anemia (%)**	519 (54.46)	219 (57.48)	300 (52.45)	0.14	0.16
**Electrolyte Abnormality (%)**	583 (62.49)	268 (73.42)	315 (55.46)	<0.001*	<0.001*
**Inflammation (%)**	597 (62.78)	325 (85.30)	272 (47.72)	<0.001*	<0.001*
**Oxygen Exchange Abnormality (%)**	604 (64.26)	226 (59.16)	378 (67.74)	0.009*	0.03*
**Acid–Base Abnormality (%)**	629 (69.97)	273 (73.39)	356 (67.55)	0.07	0.12

Note: The data are the numbers of patients, with percentages in parentheses. Adjusted *p*-values were corrected for multiple comparisons using the Benjamini–Hochberg procedure.

Renal abnormalities were defined as elevated blood urea nitrogen and creatinine. Hepatic abnormalities included elevated alanine aminotransferase and aspartate aminotransferase. Cardiac abnormalities were identified by elevated creatine kinase or creatine kinase myocardial band. Electrolyte abnormalities included deviations in plasma chloride, calcium, sodium, potassium, or magnesium levels. Inflammation was defined as elevated C-reactive protein, white blood cell count, neutrophils, or reduced lymphocytes. Anemia was indicated by reduced red blood cell count, hemoglobin, or hematocrit levels. Acid–base abnormalities included deviations in arterial blood pH, lactate, partial pressure of carbon dioxide, actual base excess, bicarbonate, or anion gap. Coagulation abnormalities were defined as reduced platelets, low platelet ratio, mean platelet volume, or plateletcrit. Oxygen exchange abnormalities included reduced arterial oxygen partial pressure or arterial oxygen saturation. The normal reference ranges for the pediatric laboratory values listed above were defined according to two industry standards.

2.6. Supplementary Table S6. Comparison of Laboratory Test Results Between Subclasses in the Whole Cohort.

	Overall	Cluster 1	Cluster 2	*p*	Adjusted *p*
**Laboratory Tests**	1116	446	670		
**Patients with Hepatic Function Tests**	931 (83.42)	364 (81.61)	567 (84.63)		
**α-HBDH, U/L (median [IQR])**	206.00 [177.00,256.00]	232.00 [192.75, 286.50]	195.00 [170.00, 232.00]	<0.001*	<0.001*
**ALT, U/L (median [IQR])**	23.75 [16.80,36.25]	19.70 [14.20, 29.00]	26.50 [19.40, 41.20]	<0.001*	<0.001*
**LDH, U/L (median [IQR])**	290.00 [251.00,348.00]	315.50 [266.75, 388.00]	279.00 [245.00, 326.00]	<0.001*	<0.001*
**AST, U/L (median [IQR])**	37.15 [29.30,48.58]	35.60 [28.40, 44.60]	39.00 [30.20, 51.40]	0.005*	0.007*
**TP, g/L (mean (SD))**	57.10 [53.40,61.10]	58.80 [54.20, 63.20]	56.40 [52.88, 59.80]	<0.001*	<0.001*
**Albumin, g/L (median [IQR])**	39.50 [36.70,41.80]	39.60 [36.40, 42.10]	39.50 [36.90, 41.60]	0.85	0.87
**TBil, µmol/L (median [IQR])**	8.90 [5.50,16.70]	6.20 [4.20, 8.83]	12.75 [7.50, 23.05]	<0.001*	<0.001*
**Globulin, g/L (median [IQR])**	17.50 [15.30,20.30]	19.10 [16.40, 21.90]	16.80 [14.70, 19.00]	<0.001*	<0.001*
**A/G Ratio (median [IQR])**	2.20 [1.90,2.60]	2.10 [1.80, 2.40]	2.40 [2.00, 2.70]	<0.001*	<0.001*
**DBil, µmol/L (median [IQR])**	2.30 [1.40,4.10]	1.55 [1.00, 2.30]	3.20 [1.90, 4.80]	<0.001*	<0.001*
**Amylase, U/L (median [IQR])**	12.00 [7.00,21.00]	19.00 [11.00, 29.25]	10.00 [6.00, 15.00]	<0.001*	<0.001*
**Patients with Renal Function Tests**	933 (83.60)	365 (81.84)	568 (84.78)		
**BUN, mmol/L (median [IQR])**	2.70 [1.93,3.50]	2.90 [2.10, 3.70]	2.57 [1.87, 3.40]	<0.001*	<0.001*
**Creatinine, µmol/L (median [IQR])**	20.30 [17.30,23.60]	20.90 [17.10, 24.30]	20.15 [17.38, 23.02]	0.22	0.26
**Patients with Cardiac Function Tests**	931 (83.42)	364 (81.61)	567 (84.63)		
**CK, U/L (median [IQR])**	94.00 [64.00,145.00]	86.50 [54.00, 153.00]	97.50 [71.00, 142.25]	0.003*	0.004*
**CK-MB, ng/mL (median [IQR])**	4.14 [2.26,11.00]	3.38 [1.50, 9.00]	4.60 [2.70, 11.85]	<0.001*	<0.001*

**Patients with Electrolyte Tests**	933 (83.60)	365 (83.89)	568 (83.41)		
**Cl^-, mmol/L (median [IQR])**	102.00 [100.00,104.00]	102.00 [99.00, 104.00]	102.00 [100.00, 104.00]	0.11	0.14
**Ca^{2+}, mmol/L (mean (SD))**	2.42 [2.30,2.52]	2.32 [2.23, 2.42]	2.47 [2.39, 2.57]	<0.001*	<0.001*
**Na^+, mmol/L (median [IQR])**	138.00 [136.00,140.00]	138.00 [136.00, 140.00]	138.00 [136.00, 140.00]	0.14	0.18
**K^+, mmol/L (median [IQR])**	4.84 (0.63)	4.55 (0.61)	5.02 (0.58)	<0.001*	<0.001*
**Mg^{2+}, mmol/L (median [IQR])**	0.93 [0.87,1.01]	0.98 [0.90, 1.09]	0.91 [0.84, 0.97]	<0.001*	<0.001*
**Patients with Inflammation Tests**	952 (85.30)	381 (85.43)	571 (85.22)		
**CRP, mg/L (median [IQR])**	2.98 [1.00,9.00]	7.00 [2.00, 23.93]	1.00 [0.48, 4.00]	<0.001*	<0.001*
**WBC, $\times10^9$/L (median [IQR])**	9.29 [7.04,12.09]	10.15 [7.01, 13.39]	8.93 [7.05, 11.32]	<0.001*	<0.001*
**Neutrophils, $\times10^9$/L (median [IQR])**	3.40 [2.08,5.64]	5.44 [3.26, 8.19]	2.56 [1.73, 4.00]	<0.001*	<0.001*
**Monocytes, $\times10^9$/L (median [IQR])**	0.72 [0.50,1.06]	0.65 [0.40, 1.07]	0.75 [0.55, 1.04]	<0.001*	0.001*
**Basophils, $\times10^9$/L (median [IQR])**	0.01 [0.01,0.03]	0.01 [0.01, 0.02]	0.02 [0.01, 0.03]	<0.001*	<0.001*
**Eosinophils, $\times10^9$/L (median [IQR])**	0.06 [0.01,0.21]	0.01 [0.00, 0.08]	0.12 [0.03, 0.26]	<0.001*	<0.001*
**Lymphocytes, $\times10^9$/L (median [IQR])**	4.36 [2.73,6.05]	3.03 [1.92, 4.98]	5.00 [3.70, 6.40]	<0.001*	<0.001*
**Patients with Anemia Tests**	953 (85.39)	381 (85.43)	572 (85.37)		
**RBC, $\times10^{12}$/L (mean (SD))**	3.72 [3.31,4.17]	4.00 [3.59, 4.37]	3.57 [3.23, 4.01]	<0.001*	<0.001*
**HCT, % (median [IQR])**	0.32 [0.29,0.34]	0.32 [0.29, 0.35]	0.32 [0.29, 0.34]	0.16	0.19
**HGB, g/L (median [IQR])**	105.00 [97.00,115.00]	105.00 [97.00, 114.00]	106.00 [96.75, 115.25]	0.47	0.53
**MCHC, g/L (median [IQR])**	332.00 [324.00,341.00]	329.00 [320.00, 337.00]	335.00 [326.75, 343.25]	<0.001*	<0.001*
**MCH, pg (median [IQR])**	28.70 [26.50,30.70]	26.70 [25.40, 28.50]	29.80 [28.00, 31.40]	<0.001*	<0.001*

**MCV, fL (mean (SD))**	86.00 [80.50,91.40]	81.20 [76.70, 86.50]	88.75 [84.00, 93.10]	<0.001*	<0.001*
**Patients with Coagulation Function Tests**	951 (85.22)	380 (85.20)	571 (85.22)		
**Platelets, ×10^9/L (median [IQR])**	393.00 [313.00,495.00]	356.00 [284.00, 451.00]	415.00 [336.75, 517.25]	<0.001*	<0.001*
**LPR (median [IQR])**	0.24 [0.20,0.30]	0.23 [0.19, 0.29]	0.24 [0.20, 0.30]	0.01*	0.01*
**MPV, fL (median [IQR])**	10.00 [9.40,10.70]	9.90 [9.30, 10.60]	10.10 [9.50, 10.70]	0.003*	0.005*
**Plateletcrit, % (median [IQR])**	0.39 [0.32,0.49]	0.35 [0.28, 0.45]	0.42 [0.35, 0.51]	<0.001*	<0.001*
**Patients with Oxygen Exchange Tests**	940 (84.23)	382 (85.65)	558 (83.28)		
**PaO_2, mmHg (median [IQR])**	72.40 [63.90,88.10]	75.10 [65.05, 91.73]	71.00 [63.20, 85.60]	0.002*	0.003*
**SaO_2, % (median [IQR])**	95.60 [93.30,97.40]	95.70 [93.10, 97.50]	95.55 [93.50, 97.38]	0.97	0.98
**Patients with Acid–Base Balance Tests**	908 (81.36)	374 (83.86)	534 (79.70)		
**pH (median [IQR])**	7.39 [7.35,7.41]	7.38 [7.35, 7.41]	7.39 [7.36, 7.42]	0.17	0.21
**Lactate, mmol/L (median [IQR])**	1.50 [1.10,2.00]	1.30 [1.00, 1.65]	1.70 [1.20, 2.20]	<0.001*	<0.001*
**pCO_2, mmHg (median [IQR])**	39.45 [35.27,44.40]	38.40 [34.70, 42.50]	40.55 [35.60, 45.70]	<0.001*	<0.001*
**ABE (median [IQR])**	-1.00 [-2.92,1.00]	-1.90 [-3.70, 0.30]	-0.50 [-2.50, 1.20]	<0.001*	<0.001*
**HCO_3^-, mmol/L (median [IQR])**	23.20 [21.10,25.30]	22.35 [20.67, 24.40]	23.70 [21.80, 25.80]	<0.001*	<0.001*
**AG, mmol/L (median [IQR])**	8.00 [4.80,10.50]	9.40 [7.00, 11.60]	6.60 [3.90, 9.60]	<0.001*	<0.001*

Note: Unless otherwise indicated, data are presented as the number of patients, with percentages in parentheses. Adjusted *p*-values were corrected for multiple comparisons using the Benjamini–Hochberg procedure.

The following abbreviations are used in the table. SD: Standard Deviation, IQR: Interquartile Range, α-HBDH: alpha-hydroxybutyrate dehydrogenase, ALT: alanine aminotransferase, LDH: lactate dehydrogenase, AST: aspartate aminotransferase, TP: total protein, TBil: total bilirubin, A/G Ratio: albumin-to-globulin ratio, DBil: direct bilirubin, BUN: blood urea nitrogen, Cl^-: serum chloride ion, Ca^{2+}: serum calcium ion, Na^+: serum sodium ion, K^+: serum potassium ion, Mg^{2+}: serum magnesium ion, RBC: red blood cell count, HCT: hematocrit, HGB: hemoglobin, MCHC: mean corpuscular hemoglobin concentration, MCH: mean corpuscular hemoglobin, MCV: mean corpuscular volume, LPR: low platelet ratio, MPV: mean platelet volume, PaO_2: arterial oxygen partial pressure, SaO_2:

arterial oxygen saturation, pCO_2: partial pressure of carbon dioxide, ABE: actual base excess, HCO_3^-: bicarbonate, AG: anion gap.

2.7. Supplementary Table S7. Comparison of Laboratory Test Abnormalities Between Subclasses in the Mechanical Ventilation Group.

	Overall	Cluster 1	Cluster 2	*p*	Adjusted *p*
	368	159	209		
**Renal Abnormality (%)**	21 (6.86)	11 (8.15)	10 (5.85)	0.57	0.76
**Hepatic Abnormality (%)**	41 (13.40)	13 (9.63)	28 (16.37)	0.12	0.36
**Coagulation Abnormality (%)**	62 (19.56)	26 (18.44)	36 (20.45)	0.76	0.86
**Anemia (%)**	120 (37.85)	59 (41.84)	61 (34.66)	0.23	0.52
**Cardiac Abnormality (%)**	173 (56.54)	59 (43.70)	114 (66.67)	<0.001*	<0.001*
**Oxygen Exchange Abnormality (%)**	198 (61.88)	86 (59.72)	112 (63.64)	0.55	0.76
**Acid–Base Abnormality (%)**	234 (74.29)	105 (74.47)	129 (74.14)	1.00	1.00
**Electrolyte Abnormality (%)**	237 (77.45)	107 (79.26)	130 (76.02)	0.59	0.76
**Inflammation (%)**	242 (76.34)	131 (92.91)	111 (63.07)	<0.001*	<0.001*

Note: The data are the numbers of patients, with percentages in parentheses. Adjusted *p*-values were corrected for multiple comparisons using the Benjamini–Hochberg procedure.

Renal abnormalities were defined as elevated blood urea nitrogen and creatinine. Hepatic abnormalities included elevated alanine aminotransferase and aspartate aminotransferase. Cardiac abnormalities were identified by elevated creatine kinase or creatine kinase myocardial band. Electrolyte abnormalities included deviations in plasma chloride, calcium, sodium, potassium, or magnesium levels. Inflammation was defined as elevated C-reactive protein, white blood cell count, neutrophils, or reduced lymphocytes. Anemia was indicated by reduced red blood cell count, hemoglobin, or hematocrit levels. Acid–base abnormalities included deviations in arterial blood pH, lactate, partial pressure of carbon dioxide, actual base excess, bicarbonate, or anion gap. Coagulation abnormalities were defined as reduced platelets, low platelet ratio, mean platelet volume, or plateletcrit. Oxygen exchange abnormalities included reduced arterial oxygen partial pressure or arterial oxygen saturation. The normal reference ranges for the pediatric laboratory values listed above were defined according to two industry standards.

2.8. Supplementary Table S8. Comparison of Laboratory Test Results Between Subclasses in the Mechanical Ventilation Group.

	Overall	Cluster 1	Cluster 2	*p*	Adjusted *p*
**Laboratory Tests**	368	159	209		
**Patients with Hepatic Function Tests**	306 (83.15)	135 (84.91)	171 (81.82)		
**α-HBDH, U/L (median [IQR])**	242.00 [200.25,299.75]	255.00 [203.00, 315.50]	236.00 [198.00, 286.00]	0.03*	0.06
**ALT, U/L (median [IQR])**	21.95 [15.72,35.67]	17.70 [13.35, 25.75]	25.80 [19.05, 43.60]	<0.001*	<0.001*
**LDH, U/L (median [IQR])**	325.00 [276.50,395.00]	330.00 [275.50, 415.50]	318.00 [279.00, 376.50]	0.22	0.32
**AST, U/L (median [IQR])**	37.85 [31.63,48.32]	35.90 [29.95, 43.10]	41.80 [32.20, 54.45]	<0.001*	0.002*
**TP, g/L (mean (SD))**	56.85 (5.81)	57.67 (5.73)	56.21 (5.81)	0.03*	0.06
**Albumin, g/L (median [IQR])**	38.55 [35.40,40.90]	38.60 [35.40, 41.00]	38.40 [35.55, 40.85]	0.89	0.96
**TBil, µmol/L (median [IQR])**	7.20 [4.60,11.85]	6.20 [4.25, 8.95]	8.60 [5.65, 14.50]	<0.001*	<0.001*
**Globulin, g/L (median [IQR])**	18.20 [15.70,21.48]	19.30 [16.50, 22.05]	17.80 [15.45, 20.40]	0.004*	0.01*
**A/G Ratio (median [IQR])**	2.10 [1.80,2.40]	2.00 [1.80, 2.30]	2.20 [1.80, 2.50]	0.04*	0.08
**DBil, µmol/L (median [IQR])**	2.00 [1.20,3.00]	1.60 [1.05, 2.45]	2.20 [1.40, 3.65]	<0.001*	0.002*
**Amylase, U/L (median [IQR])**	14.00 [8.25,26.00]	21.00 [12.00, 34.00]	11.00 [7.00, 18.50]	<0.001*	<0.001*
**Patients with Renal Function Tests**	306 (83.15)	135 (84.91)	171 (81.82)		
**BUN, mmol/L (median [IQR])**	2.99 [2.29,3.90]	3.02 [2.38, 3.92]	2.96 [2.20, 3.84]	0.19	0.28
**Creatinine, µmol/L (median [IQR])**	21.00 [18.00,24.70]	21.60 [18.60, 25.30]	20.50 [17.90, 24.45]	0.20	0.29
**Patients with Cardiac Function Tests**	306 (83.15)	96 (78.05)	210 (85.71)		
**CK, U/L (median [IQR])**	97.50 [61.00,160.25]	83.00 [57.00, 119.50]	114.00 [65.50, 171.00]	<0.001*	0.001*
**CK-MB, ng/mL (median [IQR])**	5.14 [2.16,12.92]	3.40 [1.42, 9.13]	6.73 [3.28, 13.10]	<0.001*	<0.001*
**Patients with Electrolyte Tests**	306 (83.15)	96 (78.05)	210 (85.71)		
**Cl^-, mmol/L (median [IQR])**	101.00 [99.00,104.00]	101.00 [99.00, 104.00]	101.00 [99.00, 104.00]	0.98	

**Ca^{2+}, mmol/L (mean (SD))**	2.33 (0.16)	2.29 (0.13)	2.37 (0.18)	<0.001*	<0.001*
**Na^{+}, mmol/L (median [IQR])**	139.00 [136.00,141.00]	139.00 [137.00, 141.00]	139.00 [136.00, 140.00]	0.19	0.28
**K^{+}, mmol/L (median [IQR])**	4.82 [4.33,5.25]	4.72 [4.19, 5.03]	4.95 [4.50, 5.36]	<0.001*	0.002*
**Mg^{2+}, mmol/L (median [IQR])**	0.98 [0.90,1.09]	1.01 [0.92, 1.12]	0.97 [0.89, 1.06]	0.01*	0.03*
**Patients with Inflammation Tests**	317 (86.14)	141 (88.68)	176 (84.21)		
**CRP, mg/L (median [IQR])**	4.00 [1.00,11.00]	6.00 [2.00, 16.87]	2.10 [1.00, 8.03]	<0.001*	<0.001*
**WBC, $\times10^{9}$/L (median [IQR])**	9.36 [7.01,12.60]	10.29 [7.21, 12.75]	8.95 [6.88, 12.41]	0.048*	0.10
**Neutrophils, $\times10^{9}$/L (median [IQR])**	4.31 [2.59,6.85]	6.09 [4.14, 8.19]	3.38 [2.12, 5.18]	<0.001*	<0.001*
**Monocytes, $\times10^{9}$/L (median [IQR])**	0.74 [0.46,1.15]	0.65 [0.40, 1.16]	0.79 [0.53, 1.12]	0.05	0.10
**Basophils, $\times10^{9}$/L (median [IQR])**	0.01 [0.01,0.03]	0.01 [0.01, 0.02]	0.02 [0.01, 0.03]	0.07	0.12
**Eosinophils, $\times10^{9}$/L (median [IQR])**	0.01 [0.00,0.08]	0.01 [0.00, 0.04]	0.03 [0.01, 0.12]	<0.001*	<0.001*
**Lymphocytes, $\times10^{9}$/L (median [IQR])**	3.29 [2.09,5.04]	2.55 [1.85, 4.00]	4.12 [2.56, 5.48]	<0.001*	<0.001*
**Patients with Anemia Tests**	317 (86.14)	141 (88.68)	176 (84.21)		
**RBC, $\times10^{12}$/L (mean (SD))**	3.86 (0.60)	3.96 (0.57)	3.79 (0.62)	0.01*	0.03*
**HCT, % (median [IQR])**	0.32 [0.29,0.35]	0.32 [0.29, 0.35]	0.32 [0.29, 0.35]	0.67	0.78
**HGB, g/L (median [IQR])**	106.00 [96.00,115.00]	105.00 [95.00, 114.00]	107.50 [96.00, 117.00]	0.10	0.16
**MCHC, g/L (median [IQR])**	330.00 [322.00,339.00]	326.00 [317.00, 336.00]	333.00 [325.75, 339.25]	<0.001*	<0.001*
**MCH, pg (median [IQR])**	27.80 [25.80,30.20]	26.70 [25.00, 28.40]	29.00 [26.70, 30.92]	<0.001*	<0.001*
**MCV, fL (mean (SD))**	84.04 (8.53)	81.36 (8.34)	86.18 (8.07)	<0.001*	<0.001*
**Patients with Coagulation Function Tests**	317 (86.14)	141 (88.68)	176 (84.21)		
**Platelets, $\times10^{9}$/L (median [IQR])**	371.00 [304.00,470.00]	366.00 [298.00, 455.00]	383.00 [308.25, 505.50]	0.15	0.23
**LPR (median [IQR])**	0.24 [0.20,0.29]	0.23 [0.19, 0.28]	0.24 [0.20, 0.30]	0.12	0.19

**MPV, fL (median [IQR])**	10.00 [9.40,10.60]	10.00 [9.30, 10.50]	10.00 [9.40, 10.70]	0.13	0.21
**Plateletcrit, % (median [IQR])**	0.38 [0.31,0.47]	0.36 [0.29, 0.45]	0.39 [0.32, 0.50]	0.03*	0.07
**Patients with Oxygen Exchange Tests**	320 (86.96)	144 (90.57)	176 (84.21)		
**PaO_2, mmHg (median [IQR])**	73.85 [63.55,91.12]	75.45 [63.48, 89.80]	72.70 [63.55, 92.23]	0.51	0.61
**SaO_2, % (median [IQR])**	95.50 [92.97,97.40]	95.50 [92.40, 97.23]	95.50 [93.30, 97.50]	0.33	0.44
**Patients with Acid–Base Balance Tests**	316 (85.87)	142 (89.31)	174 (83.25)		
**pH (median [IQR])**	7.38 [7.34,7.40]	7.37 [7.34, 7.40]	7.38 [7.35, 7.41]	0.09	0.14
**Lactate, mmol/L (median [IQR])**	1.20 [0.90,1.60]	1.10 [0.90, 1.40]	1.20 [0.90, 1.70]	0.06	0.1
**pCO_2, mmHg (median [IQR])**	41.40 [38.00,46.80]	40.30 [37.30, 44.50]	43.55 [38.68, 47.92]	<0.001*	0.002*
**ABE (median [IQR])**	-0.50 [-2.60,1.52]	-1.60 [-3.65, 0.60]	0.25 [-1.67, 2.60]	<0.001*	<0.001*
**HCO_3^- (median [IQR])**	23.90 [21.78,26.30]	22.80 [21.28, 25.10]	24.90 [22.67, 27.22]	<0.001*	<0.001*
**AG, mmol/L (median [IQR])**	8.00 [5.22,10.10]	9.40 [8.00, 11.60]	6.10 [3.60, 9.10]	<0.001*	<0.001*

Note: Unless otherwise indicated, data are presented as the number of patients, with percentages in parentheses. Adjusted *p*-values were corrected for multiple comparisons using the Benjamini–Hochberg procedure.

The following abbreviations are used in the table. SD: Standard Deviation, IQR: Interquartile Range, α-HBDH: alpha-hydroxybutyrate dehydrogenase, ALT: alanine aminotransferase, LDH: lactate dehydrogenase, AST: aspartate aminotransferase, TP: total protein, TBil: total bilirubin, A/G Ratio: albumin-to-globulin ratio, DBil: direct bilirubin, BUN: blood urea nitrogen, Cl^-: serum chloride ion, Ca^{2+}: serum calcium ion, Na^+: serum sodium ion, K^+: serum potassium ion, Mg^{2+}: serum magnesium ion, RBC: red blood cell count, HCT: hematocrit, HGB: hemoglobin, MCHC: mean corpuscular hemoglobin concentration, MCH: mean corpuscular hemoglobin, MCV: mean corpuscular volume, LPR: low platelet ratio, MPV: mean platelet volume, PaO_2: arterial oxygen partial pressure, SaO_2: arterial oxygen saturation, pCO_2: partial pressure of carbon dioxide, ABE: actual base excess, HCO_3^-: bicarbonate, AG: anion gap.

2.9. Supplementary Table S9. Etiological Comparison among Subclasses within the Mechanical Ventilation Cohort.

	Overall	Cluster 1	Cluster 2	*p*	Adjusted *p*
	368	159	209		
**Viral Infection (%)**	183 (49.73)	70 (44.03)	113 (54.07)	0.07	0.25
**Influenza B (%)**	2 (0.65)	2 (1.46)	0 (0.00)	0.39	0.64
**Parainfluenza virus (%)**	21 (6.86)	8 (5.84)	13 (7.69)	0.68	0.93
**Respiratory syncytial virus (%)**	141 (46.08)	50 (36.50)	91 (53.85)	0.004*	0.02*
**Influenza A (%)**	17 (5.56)	7 (5.11)	10 (5.92)	0.96	1.00
**Adenovirus (%)**	19 (6.21)	9 (6.57)	10 (5.92)	1.00	1.00
**Atypical Infection (%)**	22 (7.58)	11 (6.92)	11 (5.26)	0.66	0.93
***Mycoplasma pneumoniae* (%)**	21 (7.22)	10 (7.41)	11 (7.05)	1.00	1.00
***Chlamydophila pneumoniae* (%)**	1 (0.36)	1 (0.78)	0 (0.00)	0.93	1.00
**Bacterial Infection (%)**	142 (38.59)	63 (39.62)	79 (37.80)	0.80	1.00
***Escherichia coli* (%)**	14 (4.28)	6 (4.14)	8 (4.40)	1.00	1.00
***Haemophilus influenzae* (%)**	28 (8.56)	18 (12.41)	10 (5.49)	0.04*	0.12
***Klebsiella pneumoniae* (%)**	40 (12.23)	17 (11.72)	23 (12.64)	0.94	1.00
***Streptococcus pneumoniae* (%)**	38 (11.62)	18 (12.41)	20 (10.99)	0.82	1.00
***Staphylococcus aureus* (%)**	40 (12.23)	16 (11.03)	24 (13.19)	0.67	0.93
***Pseudomonas aeruginosa* (%)**	8 (2.45)	3 (2.07)	5 (2.75)	0.97	1.00
***Acinetobacter baumannii* (%)**	18 (5.52)	8 (5.56)	10 (5.49)	1.00	1.00

Note: Except where indicated, the data are the numbers of patients, with percentages in parentheses. Adjusted *p*-values were corrected for multiple comparisons using the Benjamini–Hochberg procedure. * denotes $p < 0.05$.

2.10. Supplementary Table S10. Age-Stratified Comparison of Etiological Distribution in the Whole Cohort.

	Overall	Age < 150 days	Age ≥ 150 days	*p*	Adjusted *p*
	1116	771	345		
**Viral Infection (%)**	499 (44.71)	362 (46.95)	137 (39.71)	0.03*	0.046*
**Influenza B (%)**	10 (1.06)	7 (1.08)	3 (1.01)	1.00	1.00
**Parainfluenza virus (%)**	97 (10.28)	68 (10.53)	29 (9.73)	0.80	0.92
**Respiratory syncytial virus (%)**	365 (38.67)	283 (43.81)	82 (27.52)	<0.001*	<0.001*
**Influenza A (%)**	24 (2.54)	10 (1.55)	14 (4.70)	0.01*	0.02*
**Adenovirus (%)**	33 (3.50)	10 (1.55)	23 (7.72)	<0.001*	<0.001*
**Atypical Infection (%)**	56 (6.20)	16 (2.64)	40 (13.52)	<0.001*	<0.001*
***Mycoplasma pneumoniae* (%)**	54 (5.98)	15 (2.47)	39 (13.18)	<0.001*	<0.001*
***Chlamydophila pneumoniae* (%)**	2 (0.23)	1 (0.17)	1 (0.34)	1.00	1.00
**Bacterial Infection (%)**	433 (38.80)	279 (36.19)	154 (44.64)	0.01*	0.02*
***Escherichia coli* (%)**	38 (4.02)	32 (4.94)	6 (2.02)	0.052	0.078
***Haemophilus influenzae* (%)**	96 (10.07)	50 (7.67)	46 (15.28)	<0.001*	0.001*
***Klebsiella pneumoniae* (%)**	94 (9.93)	63 (9.71)	31 (10.40)	0.83	0.92
***Streptococcus pneumoniae* (%)**	99 (10.45)	55 (8.49)	44 (14.72)	0.01*	0.01*
***Staphylococcus aureus* (%)**	162 (17.00)	130 (19.88)	32 (10.70)	<0.001*	0.002*
***Pseudomonas aeruginosa* (%)**	19 (2.01)	10 (1.54)	9 (3.02)	0.21	0.29
***Acinetobacter baumannii* (%)**	31 (3.28)	20 (3.09)	11 (3.72)	0.76	0.92

Note: Except where indicated, the data are the numbers of patients, with percentages in parentheses. Adjusted *p*-values were corrected for multiple comparisons using the Benjamini–Hochberg procedure. * denotes $p < 0.05$.

2.11. Supplementary Table S11. Generalized Linear Models (GLMs) validation of subgroup effectiveness under missing-data sensitivity analyses

		Cluster 1				Cluster 2			
**Cohort**	**Outcomes**	**β (log-time)**	**95%CI**	***p***	**Adjusted *p***	**β (log-time)**	**95%CI**	***p***	**Adjusted *p***
**Complete Case Analysis (n = 900)**									
**Whole Cohort**	Duration of ICU stay (Days)	-0.21	[-0.30, -0.12]	<0.001*	<0.001*	0.39	[0.30,0.48]	<0.001*	<0.001*
**Mechanically Ventilated Group**	Duration of ICU stay (Days)	-0.32	[-0.47, -0.16]	<0.001*	<0.001*	0.22	[0.12, 0.31]	<0.001*	<0.001*
	Mechanical Ventilation Duration (Hours)	-0.37	[-0.56, -0.18]	<0.001*	<0.001*	0.41	[0.29, 0.53]	<0.001*	<0.001*
**Missing Indicator Analysis (n = 1116)**									
**Whole Cohort**	Duration of ICU stay (Days)	-0.14	[-0.23, -0.05]	0.001*	0.002*	0.37	[0.29,0.45]	<0.001*	<0.001*
**Mechanically Ventilated Group**	Duration of ICU stay (Days)	-0.22	[-0.34, -0.09]	0.001*	0.002*	0.23	[0.14, 0.32]	<0.001*	<0.001*
	Mechanical Ventilation Duration (Hours)	-0.25	[-0.44, -0.06]	0.01*	0.01*	0.36	[0.24, 0.48]	<0.001*	<0.001*

Note: The table displays the adjusted β coefficients and 95% confidence intervals (CIs) from GLMs assessing the effect of glucocorticoid use. The analysis was conducted under two sensitivity scenarios: a Complete Case Analysis and a Missing Indicator Analysis. A negative β indicates a shorter duration for the outcome. P-values were adjusted for multiple comparisons using the Benjamini–Hochberg procedure. * denotes $p < 0.05$. Abbreviations: CI confidence interval, ICU: intensive care unit.

2.12. Supplementary Table S12. Characteristic analysis between suclasses based on missing-data sensitivity analysis.

	Whole Cohort				Mechanically Ventilated Group			
**Covariates**	**Cluster 1**	**Cluster 2**	***p***	**Adjusted *p***	**Cluster 1**	**Cluster 2**	***p***	**Adjusted *p***
**Complete Case Analysis (n = 900)**								
**Number of Patients**	298	602			97	205		
**Age**	251.50 [105.75, 514.00]	62.00 [46.25, 98.00]	<0.001*	<0.001*	307.00 [135.00, 520.00]	95.00 [52.00, 235.00]	<0.001*	<0.001*
**Inflammation (%)**	266 (89.26)	297 (49.34)	<0.001*	<0.001*	89 (91.75)	143 (69.76)	<0.001*	<0.001*
**CRP, mg/L (median [IQR])**	6.95 [1.85, 24.61]	1.19 [0.50, 5.00]	<0.001*	<0.001*	4.42 [1.60, 17.10]	3.00 [1.00, 9.00]	0.02*	0.02*
**WBC, ×10^9/L (median [IQR])**	11.28 [7.99, 14.86]	8.65 [6.89, 10.80]	<0.001*	<0.001*	11.02 [8.82, 12.80]	8.40 [6.50, 12.19]	<0.001*	<0.001*
**Neutrophils, ×10^9/L (median [IQR])**	6.77 [4.14, 9.08]	2.55 [1.75, 3.82]	<0.001*	<0.001*	6.42 [4.30, 8.49]	3.62 [2.24, 5.81]	<0.001*	<0.001*
**Lymphocytes, ×10^9/L (median [IQR])**	2.99 [1.91, 4.77]	4.78 [3.47, 6.30]	<0.001*	<0.001*	2.76 [1.94, 4.33]	3.60 [2.16, 5.26]	0.04*	0.04*
**Respiratory syncytial virus (%)**	77 (26.37)	272 (45.79)	<0.001*	<0.001*	32 (33.68)	102 (52.31)	0.004*	0.006*
**Missing Indicator Analysis (n = 1116)**								
Number of Patients	358	758			106	262		
**Age**	234.50 [98.25, 472.00]	61.50 [46.00, 100.75]	<0.001*	<0.001*	299.00 [139.50, 529.75]	95.00 [53.50, 239.25]	<0.001*	<0.001*
**Inflammation (%)**	317 (89.04)	280 (47.06)	<0.001*	<0.001*	91 (85.85)	151 (71.56)	0.007*	0.02*
**CRP, mg/L (median [IQR])**	7.00 [1.88, 24.00]	1.00 [0.50, 4.00]	<0.001*	<0.001*	4.05 [1.08, 11.75]	3.00 [1.00, 11.00]	0.19	0.31
**WBC, ×10^9/L (median [IQR])**	10.93 [7.08, 14.16]	8.80 [7.02, 11.00]	<0.001*	<0.001*	11.12 [8.57, 13.15]	8.48 [6.66, 12.18]	<0.001*	<0.001*

**Neutrophils, ×10^9/L (median [IQR])**	6.27 [3.77, 8.80]	2.56 [1.75, 3.82]	<0.001*	<0.001*	6.38 [4.26, 8.66]	3.63 [2.32, 5.87]	<0.001*	<0.001*
**Lymphocytes, ×10^9/L (median [IQR])**	3.00 [1.89, 4.80]	4.96 [3.62, 6.38]	<0.001*	<0.001*	2.76 [1.98, 4.49]	3.57 [2.16, 5.15]	0.09	0.16
**Respiratory syncytial virus (%)**	107 (30.66)	258 (43.36)	<0.001*	<0.001*	28 (27.45)	113 (55.39)	<0.001*	<0.001*

Note: The table presents baseline characteristics of the two identified subclassess (Cluster 1 and Cluster 2) from the missing data sensitivity analysis for both the whole cohort and the mechanically ventilated group. Unless otherwise indicated, data are presented as the number of patients, with percentages in parentheses. Adjusted p-values were corrected for multiple comparisons using the Benjamini–Hochberg procedure. Abbreviations: IQR: Interquartile Range.

2.13. Supplementary Table S13. Association between Glucocorticoids Use and Clinical Outcomes by Quartiles of Individual Treatment Effects.

Cohort	Outcomes	Quartile	β (log-time)	95%CI	*p*	Adjusted *p*
Whole Cohort	Duration of ICU stay (Days)	Quartile 1	-0.30	[-0.38, -0.21]	<0.001*	<0.001*
		Quartile 2	0.18	[0.06, 0.30]	0.002*	0.002*
		Quartile 3	0.47	[0.34, 0.60]	<0.001*	<0.001*
		Quartile 4	0.68	[0.54, 0.82]	<0.001*	<0.001*
Mechanically Ventilated Group	Duration of ICU stay (Days)	Quartile 1	-0.34	[-0.48, -0.21]	<0.001*	<0.001*
		Quartile 2	-0.13	[-0.28, 0.02]	0.09	0.11
		Quartile 3	0.22	[0.09, 0.36]	0.002*	0.003*
		Quartile 4	0.48	[0.38, 0.59]	<0.001*	<0.001*
	Mechanical Ventilation Duration (Hours)	Quartile 1	-0.41	[-0.61, -0.21]	<0.001*	<0.001*
		Quartile 2	-0.04	[-0.24, 0.16]	0.72	0.72
		Quartile 3	0.11	[-0.06, 0.29]	0.21	0.24
		Quartile 4	0.99	[0.81, 4.38]	<0.001*	<0.001*

Note: Results from multivariable generalized linear models (GLMs) assessing the association between glucocorticoid (GCs) use and clinical outcomes, stratified by quartiles of individual treatment effects (ITEs). The β coefficient represents the change in the log-transformed outcome for GCs users versus non-users. Adjusted p-values were corrected for multiple comparisons using the Benjamini–Hochberg procedure. Abbreviations: CI: confidence intervals, ICU: intensive care unit, GCs: glucocorticosteroids, * denotes $p < 0.05$.

2.14. Supplementary Table S14. Characteristic analysis across quartile-based ITE suclasses.

	Whole Cohort				Mechanically Ventilated Group			
**Covariates**	**Quartile 1**	**Quartile 4**	***p***	**Adjusted *p***	**Quartile 1**	**Quartile 4**	***p***	**Adjusted *p***
Number of Patients	279	279			92	92		
**Age**	297.00 [142.50, 533.50]	49.00 [40.00, 67.00]	<0.001*	<0.001*	309.50 [135.25, 539.25]	66.00 [47.00, 134.25]	<0.001*	<0.001*
**Inflammation (%)**	226 (91.87)	86 (34.26)	<0.001*	<0.001*	74 (91.36)	40 (51.95)	<0.001*	<0.001*
**CRP, mg/L (median [IQR])**	8.00 [2.92, 25.84]	1.00 [0.48, 3.00]	<0.001*	<0.001*	4.10 [1.40, 11.00]	2.20 [1.00, 5.00]	0.01*	0.01*
**WBC, ×10^9/L (median [IQR])**	10.85 [7.19, 13.75]	8.57 [7.07, 10.48]	<0.001*	<0.001*	11.36 [9.14, 13.32]	8.13 [6.95, 12.38]	<0.001*	<0.001*
**Neutrophils, ×10^9/L (median [IQR])**	6.42 [3.88, 9.30]	2.11 [1.58, 3.12]	<0.001*	<0.001*	6.78 [4.95, 8.73]	2.56 [1.81, 4.23]	<0.001*	<0.001*
**Lymphocytes, ×10^9/L (median [IQR])**	2.70 [1.81, 4.11]	5.24 [4.27, 6.51]	<0.001*	<0.001*	2.83 [2.05, 4.33]	4.46 [3.29, 5.74]	<0.001*	<0.001*
**Respiratory syncytial virus (%)**	70 (28.81)	114 (45.78)	<0.001*	<0.001*	23 (29.11)	42 (57.53)	<0.001*	<0.001*

Note: This table summarizes a comparative analysis of key baseline characteristics between patients in the highest (Quartile 1) and lowest (Quartile 4) quartiles of estimated glucocorticoids (GCs) treatment effect. Unless otherwise indicated, data are presented as the number of patients, with percentages in parentheses. Adjusted p-values were corrected for multiple comparisons using the Benjamini–Hochberg procedure. Abbreviations: IQR: Interquartile Range.

3. Supplementary Figures

3.1. Supplementary Figure S1. Enrollment flowchart of the study.

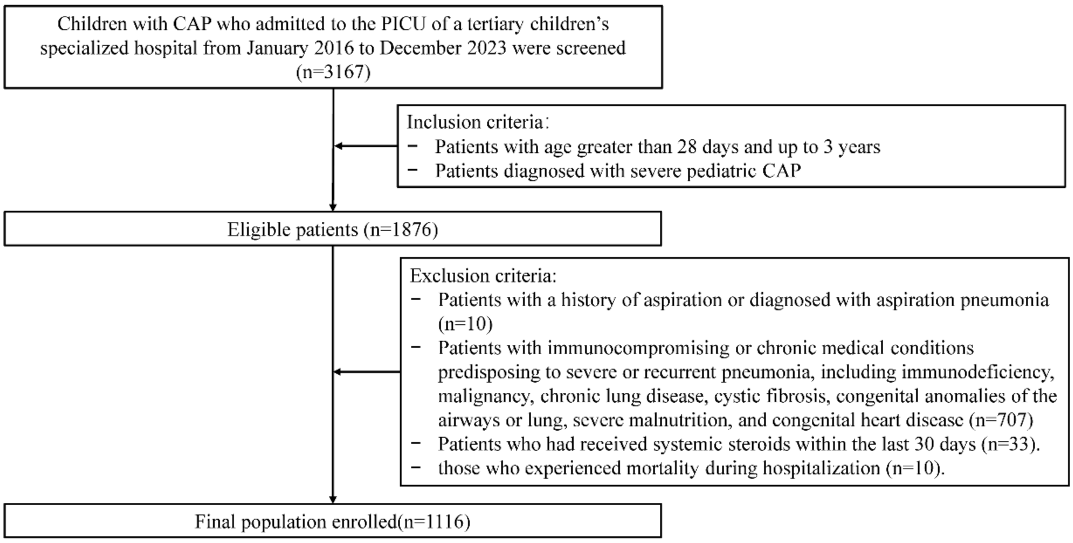

Supplementary Figure S1.

Abbreviations: CAP= Community-acquired Pneumonia. PICU= Pediatric Intensive Care Unit.

3.2. Supplementary Figure S2. Directed Acyclic Graph for Confounding Control.

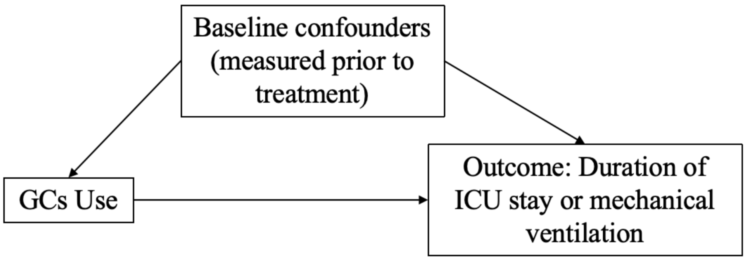

Supplementary Figure S2. The graph shows the assumed causal relationships between the treatment (GCs Use), the outcome, and the set of measured baseline confounders. Baseline confounders are a set of pre-treatment variables assumed to be common causes of both GCs use and the outcome. Abbreviations: GCs: Glucocorticoids, ICU: intensive care unit, MV: Mechanical Ventilation.

3.3. Supplementary Figure S3. Diagnostic Plots for the Causal Forest Model.

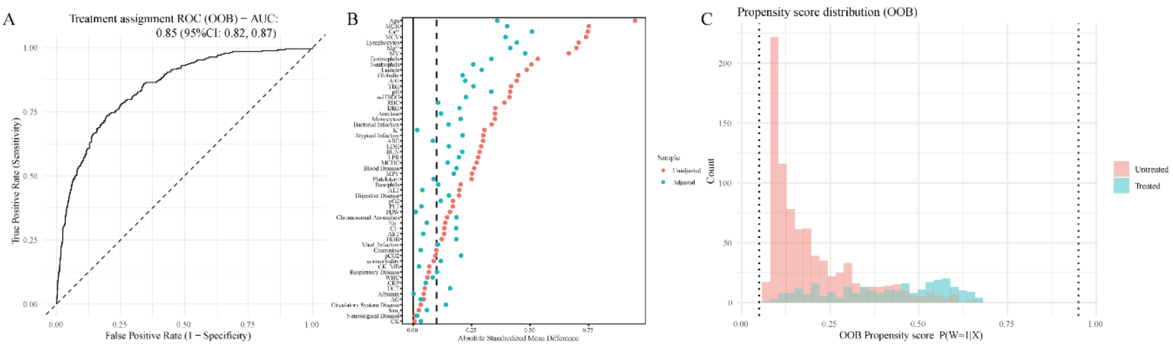

Supplementary Figure S3. (A) Out-of-Bag (OOB) Receiver Operating Characteristic (ROC) Curve and C-statistic for Treatment Assignment. (B) Covariate Balance Before and After Forest-Based Weighting. This Love plot displays the absolute standardized mean differences (SMDs) for all baseline covariates before (Unadjusted, red) and after (Adjusted, blue) inverse probability weighting derived from the causal forest model. The dashed vertical line represents |SMD| = 0.10. (C) OOB Propensity Score Overlap Distribution. This histogram illustrates the distribution of OOB propensity scores, showing the density of treated and untreated patients across the range of predicted treatment probabilities. The vertical dashed lines indicate propensity score thresholds of 0.05 and 0.95. Abreviations: α-HBDH: alpha-hydroxybutyrate dehydrogenase, ALT: alanine aminotransferase, LDH: lactate dehydrogenase, AST: aspartate aminotransferase, TP: total protein, TBil: total bilirubin, A/G: albumin-to-globulin ratio, DBil: direct bilirubin, BUN: blood urea nitrogen, Cl^-: serum chloride ion, Ca^{2+}: serum calcium ion, Na^+: serum sodium ion, K^+: serum potassium ion, Mg^{2+}: serum magnesium ion, RBC: red blood cell count, HCT: hematocrit, HGB: hemoglobin, MCHC: mean corpuscular hemoglobin concentration, MCH: mean corpuscular hemoglobin, MCV: mean corpuscular volume, LPR: low platelet ratio, MPV: mean platelet volume, PaO_2: arterial oxygen partial pressure, SaO_2: arterial oxygen saturation, pCO_2: partial pressure of carbon dioxide, ABE: actual base excess, HCO_3^-: bicarbonate, AG: anion gap.

3.4. Supplementary Figure S4. Calibration of the final causal forest models in the whole cohort and MV subgroup.

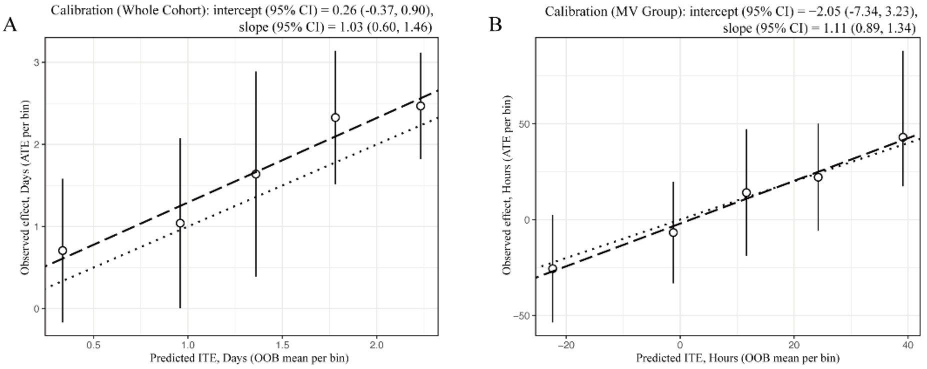

Supplementary Figure S4. Calibration plots illustrate the agreement between predicted individual treatment effects (ITEs) and estimated average treatment effects (ATEs). Patients were divided into quintiles according to predicted ITEs, and the mean observed ATE within each quintile was plotted against the mean predicted ITE. Panel A shows the calibration for the whole cohort, and Panel B for the mechanically ventilated (MV) subgroup. The dotted line represents perfect calibration (slope = 1, intercept = 0), while the dashed line indicates the fitted calibration line with 95% confidence intervals. Points closer to the identity line suggest better calibration.

3.5. Supplementary Figure S5. Characteristics of Subclasses in the Mechanical Ventilation (MV) Group.

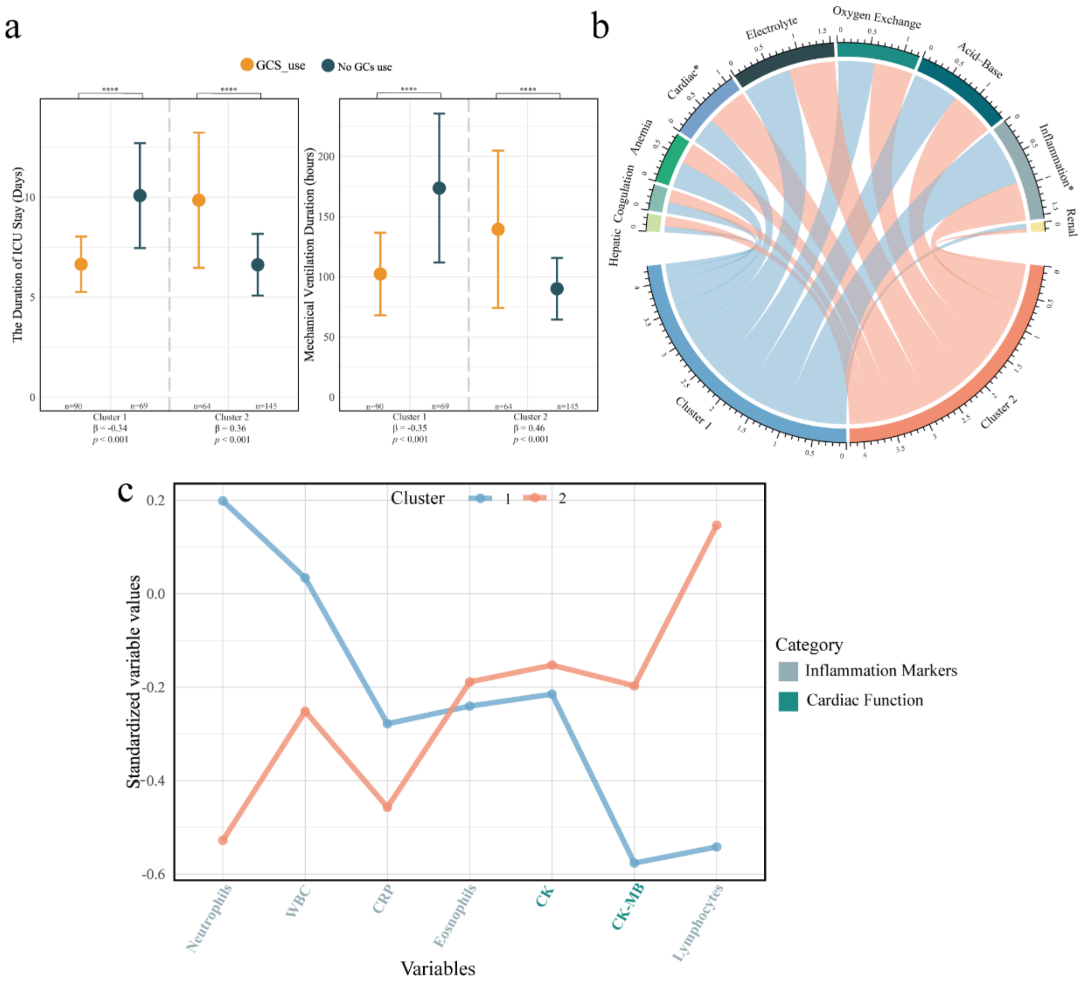

Supplementary Figure S5.

a. The dot plot with error bars comparing the duration of the intensive care unit (ICU) stay and the mechanical ventilation between glucocorticoids (GCs) users and non-users across subclasses in the MV group. The top section presents T-test results for the mechanical ventilation duration between the two groups, while the bottom section shows the adjusted β coefficients and p-values for GCs use from multivariable GLM models for each subclass, along with the number of GCs users and non-users in each subclass. "****" represents $p < 0.0001$.

b. Chord diagram showing the proportion of abnormal laboratory values across different test categories in cluster 1 and 2 among the MV group. Asterisks (*) indicate significant differences in the proportions of abnormalities between the two clusters (p<0.05).

c. The line plot depicting the median z-score–standardized values of laboratory tests in Cluster 1 and Cluster 2 within the MV group. The x-axis lists individual laboratory variables, with label colors denoting their corresponding test categories as defined in the right legend, while the y-axis represents the median z-score for each cluster. Only variables from categories with significant be-tween-cluster differences in panel (b) are shown. Z-scores were standardized across the entire cohort. Values above 0

indicate levels higher than the cohort average, whereas values below 0 indicate lower-than-average levels.

Abbreviations: GCs: Glucocorticoids, ICU: intensive care unit, WBC: white blood cell, CRP: C-reactive protein, CK: creatine kinase, CK-MB: creatine kinase myocardial band.

3.6. Supplementary Figure S6. Sensitivity analysis of cluster assignment under different missing data approaches.

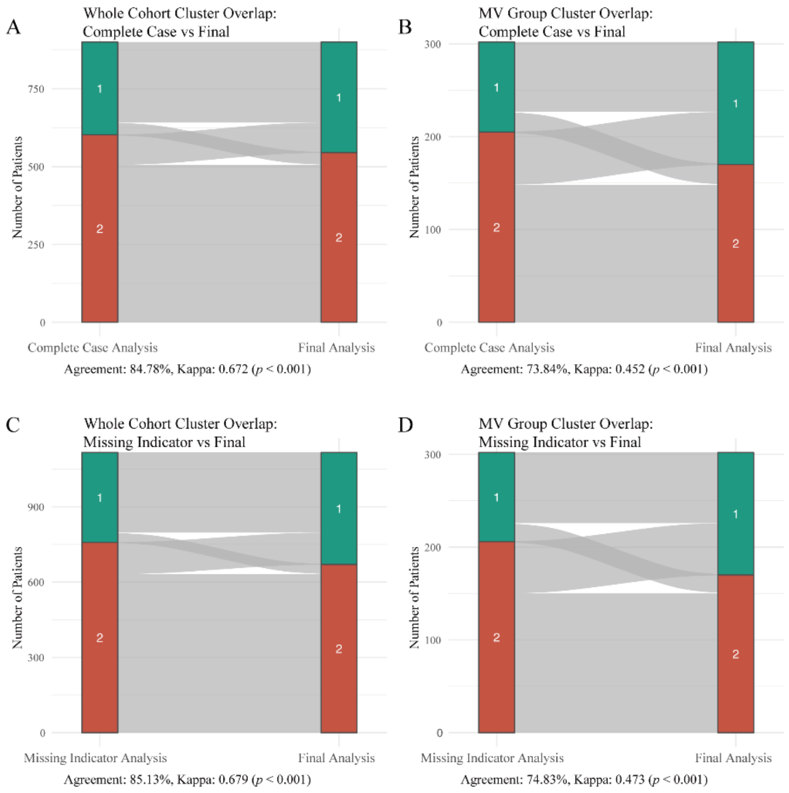

Supplementary Figure S6. Panels A–B show the concordance of cluster assignment (Cluster 1 vs. Cluster 2) between the complete-case analysis and the primary analysis for the whole cohort (A) and the mechanically ventilated (MV) subgroup (B). Panels C–D show the concordance between the missing-indicator analysis and the primary analysis for the whole cohort (C) and the MV subgroup (D). Agreement percentages and Cohen's kappa statistics are reported below each panel, indicating the overlap between sensitivity analyses and the primary analysis.

3.7. Supplementary Figure S7. Covariate balance before and after overlap weighting based on logistic regression propensity scores.

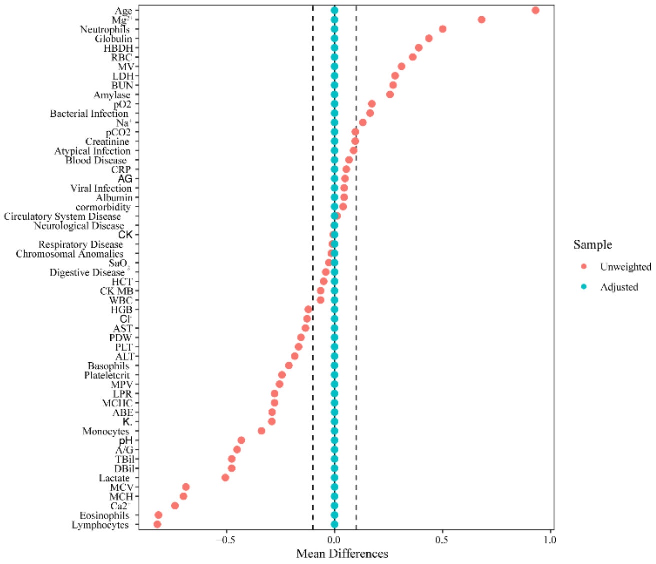

Supplementary Figure S7. Standardized mean differences (SMDs) for all baseline covariates are shown before adjustment (unweighted, red) and after overlap weighting (adjusted, blue). The dashed vertical lines represent |SMD|=0.10. Abbreviations: α-HBDH: alpha-hydroxybutyrate dehydrogenase, ALT: alanine aminotransferase, LDH: lactate dehydrogenase, AST: aspartate aminotransferase, TP: total protein, TBil: total bilirubin, A/G Ratio: albumin-to-globulin ratio, DBil: direct bilirubin, BUN: blood urea nitrogen, Cl^-: serum chloride ion, Ca^{2+}: serum calcium ion, Na^+: serum sodium ion, K^+: serum potassium ion, Mg^{2+}: serum magnesium ion, RBC: red blood cell count, HCT: hematocrit, HGB: hemoglobin, MCHC: mean corpuscular hemoglobin concentration, MCH: mean corpuscular hemoglobin, MCV: mean corpuscular volume, LPR: low platelet ratio, MPV: mean platelet volume, PaO_2: arterial oxygen partial pressure, SaO_2: arterial oxygen saturation, pCO_2: partial pressure of carbon dioxide, ABE: actual base excess, HCO_3^-: bicarbonate, AG: anion gap.
